# HER2-antigen-specific humoral immune response in breast cancer lymphocytes transplanted in hu-PBL hIL-4 NOG mice

**DOI:** 10.1038/s41598-021-92311-y

**Published:** 2021-06-17

**Authors:** Yusuke Ohno, Shino Ohshima, Asuka Miyamoto, Fuyuki Kametani, Ryoji Ito, Banri Tsuda, Yukie Kasama, Shunsuke Nakada, Hirofumi Kashiwagi, Toshiro Seki, Atsushi Yasuda, Kiyoshi Ando, Mamoru Ito, Yutaka Tokuda, Yoshie Kametani

**Affiliations:** 1 Division of Basic Medical Science, Department of Molecular Life Science, Tokai University School of Medicine, Isehara, Kanagawa 259-1193 Japan; 2 Central Institute for Experimental Animals, Kawasaki, Kanagawa 210-0821 Japan; 3 Department of Breast and Endocrine Surgery, Tokai University School of Medicine, Isehara, Kanagawa 259-1193 Japan; 4 Department of Dementia and Higher Brain Function, Tokyo Metropolitan Institute of Medical Science, Tokyo, 156-8506 Japan; 5 Department of Obstetrics and Gynecology, Tokai University School of Medicine, Isehara, Kanagawa 259-1193 Japan; 6 Division of Nephrology, Endocrinology and Metabolism, Department of Internal Medicine, Tokai University School of Medicine, Isehara, Kanagawa 259-1193 Japan; 7 Department of Hematology and Oncology, Tokai University School of Medicine, Isehara, Kanagawa 259-1193 Japan

**Keywords:** Biological techniques, Experimental organisms, Model vertebrates, Mouse

## Abstract

The status of humoral immunity of cancer patients is not clear compared to cellular immunity because the ability of specific antibody production is difficult to analyze in vitro. We previously developed a humanized mouse model to evaluate antigen-specific antibody production by transplanting human peripheral blood mononuclear cells (PBMCs) into NOG-hIL-4-Tg mice (hu-PBL hIL-4 NOG). In this study, these mice were transplanted with PBMCs derived from breast cancer patients (BC) and immunized with a human epidermal growth factor receptor 2 (HER2) peptide, CH401MAP, to analyze humoral immunity of BCs. The hu-PBL hIL-4 NOG mice recapitulated immune environment of BCs as the ratio of CD8+/CD4+T cells was lower and that of PD-1 + T cells was higher compared to healthy donors (HDs). Diverse clusters were detected in BC-mouse (BC-M) plasma components involving immunoglobulins and complements unlike HD-M, and there was a significant diversity in CH401MAP-specific IgG titers in BC-M. The number of B cell clones producing high CH401MAP-specific IgG was not increased by immunization in BC-M unlike HD-M. These results demonstrated that the humoral immunity of BCs appeared as diverse phenotypes different from HDs in hu-PBL hIL-4 NOG mice, which may provide important information for the study of personalized medicine.

## Introduction

Passive immunotherapy using antibodies against cancer antigens, such as human epidermal growth factor receptor 2 (HER2) and CD20, is considered an effective strategy for treating cancer when surgery is not feasible^1–3^. Numerous studies have indicated that effector functions, such as antibody-dependent cellular cytotoxicity induced by natural killer cells, are retained in patients undergoing passive immunotherapy. However, active immunotherapy, which induces anti-cancer antibody production in vivo, rarely induces an effective anti-cancer response^4–6^. In particular, the immune response associated with Luminal A and Luminal B subtypes with negative/low HER2 expression, which is represented by tumor-infiltrating lymphocytes, has been shown to be suppressed in breast cancer patients^7–9^. However, the mechanisms underlying this phenomenon remain unclear, mainly because the efficacy of cancer antigen-specific cellular and humoral immunity has not been elucidated in cancer patients. Although B cell-deficient mice exhibit an enhanced antitumor response^10^ and tumor-infiltrating B cells have been associated with enhanced survival in human patients^11^, the importance of B cell response remains poorly understood.

Previous studies have examined the difference in the cellularity between peripheral blood mononuclear cells (PBMCs) derived from breast cancer patients (BCs) and those derived from healthy donors (HDs)^12,13^. We reported a higher proportion of B cells and a lower proportion of CD8^+^ T cells in PBMCs derived from BCs^13,14^. In addition to transitional and naïve cells, B cells also comprise memory cells. However, it is not clear why the proportion of B cells increased; additionally, the functions of these B cells remain unknown.

Despite their limited efficacy in a subset of cancer patients, immune checkpoint antibodies have been widely used in cancer immunotherapy^15,16^. Numerous checkpoint antibodies and protocols are available for clinical application^17,18^. In particular, the blockade of the Programmed cell death 1/Programmed cell death 1 − Ligand -1 (PD-1/PD-L1) axis in PD-L1-expressing cancers has yielded remarkable results^19–22^. PD-1 expressed on the surface of activated T cells downregulates T cell receptor signaling through SHP2 activity^23^. Immune checkpoint inhibitors (ICI) prevent the interaction of PD-1-expressing T cells and PD-L1-expressing antigen-presenting cells, thereby promoting the survival of activated T cells^20^. In contrast, B cell stimulation in the humoral immune system was supported by PD-1^+^ T helper (Th) cells known as follicular helper T cells^20,24^. Therefore, PD-1 expression might be pivotal to induce effective B cell response. Although the molecular mechanism of PD-1/PD-L1 blockade in cellular immunity has been extensively investigated, there are only a few reports on PD-L1^+^ B cells and the effects of ICIs on the humoral immune system^25,26^.

The humanized mouse model is a powerful tool for the comparison of human immunity^27,28^ as the engraftment of peripheral blood (PB) cells derived from patients recapitulates their conditions^29^. We developed a second-generation humanized mouse model, hu-PBL hIL-4 NOG mouse, to assess PBMC-mediated humoral immune response in vivo^12,30^. This mouse strain was based on a severely immunodeficient NOD/Shi-scid IL2rγnull (NOG) mouse and expressed human IL-4. Human PBMCs were transplanted into the mice, after which they were immunized with the HER2 peptide, CH401MAP^31^, in combination with Freund’s Adjuvant. The high concentration of human IL-4 in circulation prevented graft-versus-host disease (GVHD) resulting from xenotransplantation and induced the production of antigen-specific human IgG antibodies. However, germinal centers and their reactions were not observed in the spleen^32^.

In this study, we aimed to compare the cellularity and functional aspects of antibody production between HDs and BCs in humanized NOG-IL-4-Tg mice, and to find the difference of immune cell population and the ability of antibody production against HER2 peptide.

## Results

### Comparison of the cellularity between healthy donor- and breast cancer patient-derived immune cells

**Figure 1:**
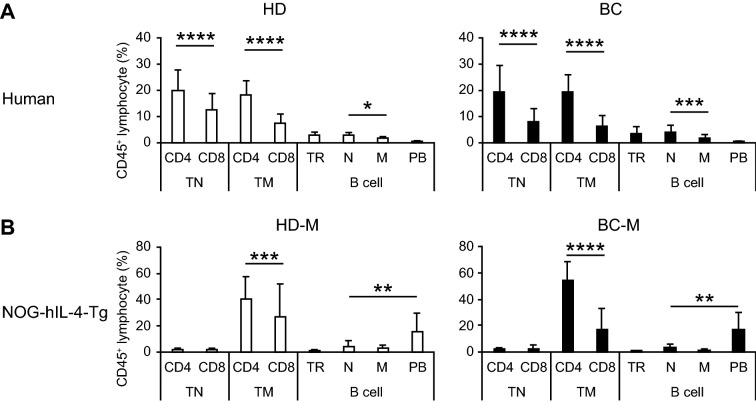
The hu-PBL hIL-4 NOG mice reflected HD/BC T cell and B cell phenotypes. Proportions of CD4 and CD8 T cells in human PBMCs and PBMC-transplanted NOG-hIL-4-Tg in human CD45-gated fractions were compared. **(A)** Healthy donor (HD) (open bar: *n* = 39) and breast cancer patient (BC) (black bar: *n* = 47) PBMCs. **(B)** NOG-hIL-4-Tg spleens transplanted with HD (filled bar: *n* = 26) or BC (white bar: *n* = 11) PBMCs. Data are expressed as mean ± Standard Deviation of each cell subset. *p = 0.05, **p = 0.01, ***p = 0.001, ****p = 0.0001. *CD4 TN* Naïve CD4^+^ T cell, *CD8 TN* Naïve CD8^+^ T cell, *CD4 TM* Memory CD4^+^ T cell, *CD8 TM* memory CD8^+^ T cell, *TR* transitional, *N* naïve, *M* memory, *P* plasmablast; *BC* breast cancer; *HD* healthy donor; *PBMCs* peripheral blood mononuclear cells.

First, we examined whether the hu-PBL hIL-4 NOG mouse replicated the immune conditions in HDs and BCs. We analyzed the proportions of T cell and B cell subsets in HD- and BC-derived PBMCs via multicolor flow cytometry (FCM) analysis. The gating strategy is shown in Fig. S1 in the Supplementary material. Fresh human PBMC and hu-PBL hIL-4 NOG mouse -engrafted human cells were analyzed using the same strategy. We found that the proportions of both naïve and memory CD4^+^ T cells were significantly higher than those of CD8^+^ T cells in both HD- and BC-derived PBMCs (Fig. 1A). Most B cells were of the transitional, naïve, or memory phenotype. On the other hand, most of the T cells in the hu-PBL hIL-4 NOG mice exhibited a memory phenotype (CD45RO^+^CD45RA^−^ fraction in Fig. S2 in the Supplementary material), while the B cells exhibited a plasmablast phenotype (CD27^+^CD38^+^ fraction in Fig. S2 in the Supplementary material), as shown in Fig. 1B. The CD8^+^ T cell/CD45^+^ cell proportion among the naïve fraction of HD-derived PBMCs tended to be higher than that among the BC-derived PBMCs (Fig. 1A, TN CD4, and CD8). On the other hand, the difference between CD4^+^ and CD8^+^ T cell proportions was larger in BC-M than in HD-M (Fig. 1B TM CD4 and CD8). The ratio of total CD8^+^/total CD4^+^ of human CD45^+^ cells was found to be 0.56 ± 0.28 in HD PBMCs, 1.08 ± 1.49 in the PBMCs of the mice transplanted with HD-PBMCs (HD-M), 0.42 ± 0.31 in BC PBMCs, and 0.41 ± 0.51 in the PBMCs of the mice transplanted with BC-PBMCs (BC-M). Thus, the CD8^+^ T cell/CD45^+^ cell proportions and the ratios of total CD8^+^/total CD4^+^ of human CD45^+^ cells were lower in BC PBMCs and BC-M, whereas CD4^+^ T cell/CD45^+^ cell proportions were not significantly different in HD-M and BC-M in total. The decrease in the CD8^+^ T cell ratio in BC PBMCs observed in the above results was similar to that reported previously^13^. These results suggest that although the maintenance of naïve lymphocyte was difficult, the hu-PBL hIL-4 NOG mouse immune system recapitulated the donors’ and patients’ memory T cell and plasmablast fractions.

As the hu-PBL hIL-4 NOG mouse partially replicated the immune conditions in HD and BC, we immunized the mice with CH401MAP and compared the immune responses in HD-M and BC-M. The total cell number in the spleen tended to be lower in BC-M than in HD-M following CH401MAP immunization. The proportion of CD45^+^ human white blood cells was comparable between HD-M and BC-M spleens (Fig. S3A in the Supplementary material). Although the total cell number was not significantly different in bone marrow (BM), the proportion of CD45^+^ cells tended to be increased in HD-M following immunization (Fig. S3B in the Supplementary material). However, no significant difference was observed in the proportions of T (CD3^+^), B (CD19^+^) and NK (CD56^+^) fractions (Fig. S3C in the Supplementary material) or CD4^+^ and CD8^+^ T cell fractions (Fig. S3D in the Supplementary material) between HD-M and BC-M.

**Figure 2:**
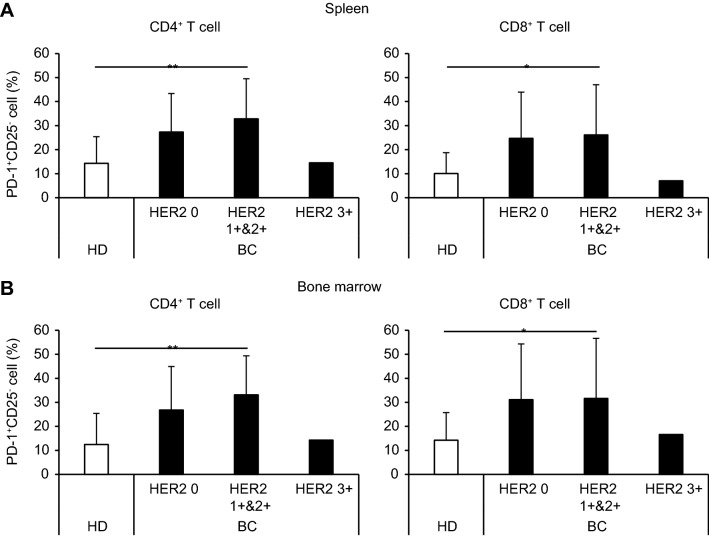
Exhaustion T cells were abundantly detected in HER2-low BC-M. Frequency of PD-1^+^CD25^−^ subset in CD4^+^ or CD8^+^ gated T cells in the spleen **(A)** and bone marrow **(B)** of NOG-hIL-4-Tg mice transplanted with HD (*n* = 22) or BC (HER2 0; *n* = 3, HER2 1 + &2 + ; *n* = 8, HER2 3 + ; *n* = 1) PBMCs. HD and BC-transplanted mice are indicated as white (HD) or black bars (BC). *p = 0.05, **p = 0.01. *BC* breast cancer; *HD* healthy donor; *HER2* Human epidermal growth factor receptor 2; *PBMCs* peripheral blood mononuclear cells.

Subsequently, we analyzed the activation/exhaustion of the T cells by examining the expression of CD25, an early activation marker, and PD-1, a late activation/exhaustion marker of T cells. Significant numbers of CD4^+^ and CD8^+^ T cells expressed CD25, PD-1, or both of the markers in HD-M and BC-M (Figs. S1A, S4C, S4D in the Supplementary Material). Among these activated T cell fractions derived from PBS- and CH401MAP-treated BC-M, the PD-1 single-positive (SP) cells tended to be abundant in both CD4^+^ and CD8^+^ T cell groups, whereas the CD25 SP and CD25/PD-1 DP fractions were comparable to HD-M in both spleen and BM. The proportion of PD-1 SP cells in CD4^+^ and CD8^+^ fractions was compared between BC-M and HD-M (Fig. 2). PD-1 SP cell ratio was significantly higher only in HER2 1 + 2 + BC-M CD4 T cells compared to HD-M. The mean fluorescence intensity (MFI) of PD-1 was found to be higher in CD4^+^ T cells than in CD8^+^ T cells in both HD-M and BC-M spleens, and there was no significant change in MFI following immunization (Fig. S4A,B in the Supplementary material). However, the proportion of PD-1^+^ cells tended to decrease among CD4^+^ and CD8^+^ T cells in BC-M spleen and BM following immunization (Fig. S4C,D in the Supplementary material). No significant difference was observed among HER2 subgroups. These results suggest that BC T cells were more prone to exhaustion compared to HD T cells, and CH401MAP immunization slightly decreased the exhaustion.

A comparable number of B cells were engrafted in the spleen and BM in HD-M and BC-M after immunization (Fig. S3C in the Supplementary material). Most of the B cells engrafted in the spleen and BM of HD-M and BC-M showed a plasmablast phenotype irrespective of immunization (Fig. S5A,B in the Supplementary material). No significant change was observed in B cell phenotype in BM and spleen in HD-M and BC-M following immunization. Moreover, B cells did not express significant levels of PD-1 (Fig. S6 in the Supplementary material).

These results suggest that the hu-PBL hIL-4 NOG mouse replicated the immune system of the donors, where the proportion of CD4^+^ T cells was higher in BC than in HD, and the T cells of HER2 0 to 2 + BC-M were significantly exhausted. However, there was no significant change in the cellularity of the engrafted human lymphocytes following immunization with the cancer antigen.

### Estimation of serum proteins in the hu-PBL hIL-4 NOG mouse

**Figure 3:**
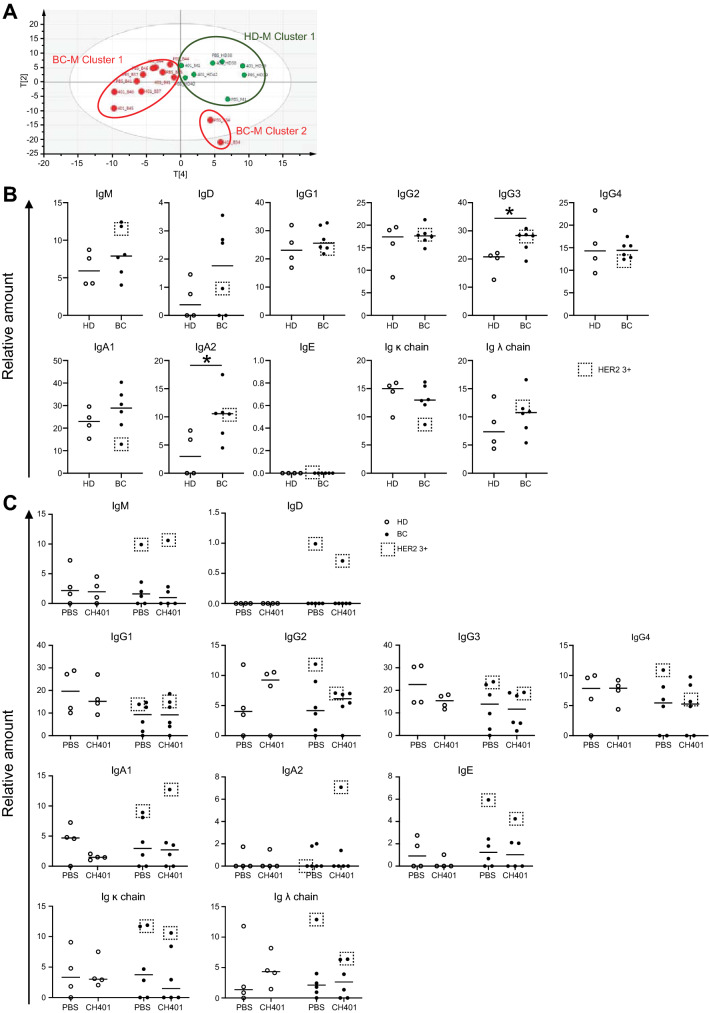
Comparison of human proteins in mouse plasma. **(A)** Principal component analysis (PCA) of human proteins in plasma of mice transplanted with HD (green, PBS; *n* = 4, CH401MAP; *n* = 4) or BC (red, PBS; *n* = 6, CH401MAP; *n* = 6). *t*-Predicted score scatterplot shows the results. The scaffold software used for PCA was SIMCA version 3 (https://www.sartorius.com/en/products/process-analytical-technology/data-analytics-software/mvda-software/simca?gclid=EAIaIQobChMI_7fm_JHY8AIVxcKWCh3A6AnCEAAYASAAEgKbEvD_BwE). Sample names are shown in Table 1. R2X[2] = 0.152; R2X[4] = 0.0974. The components are shown in Table S2. Relative amount of each human antibody class in **(B)** HD (open circle; *n* = 4) or BC (filled circle; *n* = 6) plasma and **(C)** HD-transplanted (open circle; PBS; *n* = 4, CH401MAP; *n* = 4) or BC-transplanted (filled circle; PBS; *n* = 6, CH401MAP; *n* = 6) mice. HER2 3 + BC samples in BC-Ms were labeled with dotted squares. *p = 0.05. *BC* breast cancer; *HD* healthy donor; *PBMCs* peripheral blood mononuclear cells; *PBS* phosphate-buffered saline; *Ig* immunoglobulin.

Next, we examined the humoral immunity of HD-M and BC-M. The sera of the donors and hu-PBL hIL-4 NOG mice were subjected to Liquid chromatography/Mass spectrometry (LC–MS/MS) analysis, and Principal component analysis (PCA) was performed (Fig. 3A). Although the contribution was not high, the *t*-values of p[2] and p[4] showed clear clusters among HD-M. BC-M was categorized into two clusters, BC-M clusters 1 and 2, and HD-M exhibited one large cluster (HD-M cluster) (Fig. 3A and Fig. S7A in the Supplementary material). Mice transplanted with #B37, #B41, #B44, #B45, and #B46 were grouped into the BC-M cluster 1. Mice transplanted with the PBMCs of patient #B34 (with a mucinous type of BC, more than 100 mm diameter, and HER2 2 +) belonged to BC-M cluster 2. The CH401MAP group of BC-M, especially the mice of HER2 3 + level, were observed in the low-p[2] area of the BC-M cluster 1. The components of these clusters included human immunoglobulins (Igs) and complements (Table S2 in the Supplementary material). The HD-M cluster included all four subclasses of IgG heavy chain and light chains along with various complements (pale orange boxes, which are both M1.p[4]- and M1.p[2]-positive, in Fig. S7A and Table S2 in the Supplementary material). In contrast, IgM, IgE, C1r, and C6 were included in the BC-M cluster-1 and Ig heavy chain variant 307 in BC-M cluster-2. Collectively, PCA revealed that plasma protein profiles were different in HD-M and BC-M, and the components consisted of human Igs and complement factors.

Therefore, we compared the relative amounts of the human plasma Igs and complements between HD and BC, and between HD-M and BC-M. All Ig subtypes except IgE have been detected in plasma derived from HD and BC at similar levels. However, in our data, IgG3 and IgA2 levels were significantly higher in BC plasma compared to HD plasma (Fig. 3B).

All of the subclasses were detected in the plasma of the mice (Fig. 3C). However, unlike human plasma, BC-M plasma contained lower levels of Igs. IgG1 and IgG3 levels were comparable to that of HDs and PBS-treated HD-M; however, their levels in HD-M were decreased following CH401MAP-immunization. IgE was detected in HD-M and BC-M but not in HD and BC plasmas, suggesting that high IL-4 levels affected the class switch of B cells. The decrease in IgA2 levels was also prominent in both BC-M and HD-M plasma. The BC patient submitted for LC–MS analysis (Fig. 3B) and the same donor’s BC-M (Fig. 3C) of HER2 3 + group are marked by dotted squares. While the donor sample showed high IgM level, low IgD, IgA1 and Igk levels, other classes showed intermediate levels. The BC-M sample showed highest levels in most of the Ig class or subclass, suggesting that the high antibody producibility of the patient was observed in the BC-M.

Furthermore, since the components consisted of human complements, we analyzed the expression levels of these molecules (Fig. S7 in the Supplementary material) for the plasma of donors (Fig. S7B) and hu-PBL hIL-4 NOG mice (Fig. S7C). Although all of the complements except C2 were detected in the plasma (Fig. S7B), the complements detected in the hu-PBL hIL-4 NOG mice were limited (Fig. S7C).

These results demonstrate that the plasma proteins derived from the hu-PBL hIL-4 NOG mice contained all Ig subclasses and a part of complements, although the levels are significantly different from those found in human plasma.

### Antibody production by B cells in the spleen of the hu-PBL hIL-4 NOG mouse

As the results suggest that the humoral immunity reconstituted by BC PBMCs is different from that of HD and more diverse, and that the main component is found to be human Ig, we examined the ability of B cells to secrete antigen-specific antibodies. Since breast cancer tissues often express the HER2 molecule to some extent, even if the diagnosis is not of a HER2 3 + type, we examined both the cross-reactivity to CH401MAP in BC or PBS-immunized BC-M plasma as well as the HER2 expression levels (Fig. S8 in the Supplementary material). The CH401MAP cross-reactivity in BC-M plasma was not highly correlated with HER2 expression in the patient tumor.

**Figure 4:**
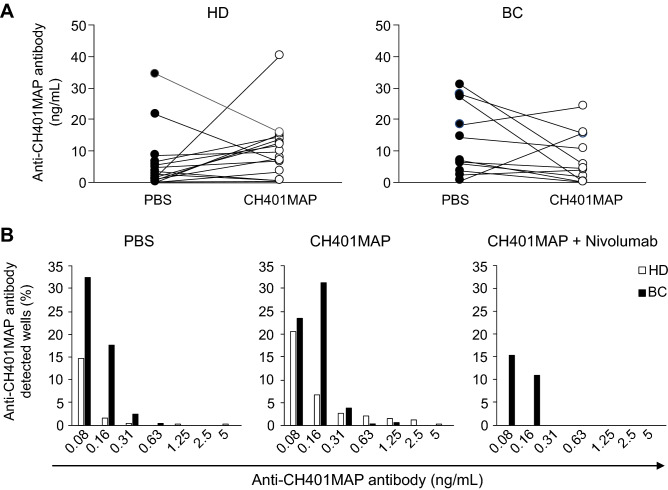
Production of specific antibody was decreased in BC immune environment. Concentrations of anti-CH401MAP antibody in plasma of HD-M and BC-M. **(A)** Transition graph of anti-CH401MAP antibody in mice transplanted with PBMCs of HD (*n* = 17) or BC (*n* = 12). The concentration was compared between PBS (filled circle) or CH401MAP (open circle) groups, and the lines represent the mice transplanted with the same donor PBMC. **(B)** Anti-CH401MAP antibody concentration in supernatant and percentage of anti-CH401MAP antibody detected in wells in a culture plate. HD- or BC-transplanted spleens were fused with P3X (*n* = 5). As the hybridoma yield is different between each experiment, positive cell ratio was calculated based on the sum of all experiments. *BC* breast cancer; *HD* healthy donor; *PBMCs* peripheral blood mononuclear cells; *PBS* phosphate-buffered saline.

Further, we compared CH401MAP-specific antibody levels in PBS-immunized and CH401MAP-immunized mice transplanted with PBMCs of the same donor. We found that the specific antibody levels tended to increase in HD-M plasma and to decrease in BC-M plasma among CH401MAP-immunized mice as a whole (Fig. 4A). Only 4/12 exhibited an increase in the specific antibody level (two HER2 3 + (2/2), one HER2 1 + Luminal A (1/7) and one HER2 0 (1/3) scirrhous patients), while the specific antibody levels were upregulated in HD-M (10/16 of mice showed an increase in the specific antibody level; a total of 5 mice showed a decrease of its level and an individual mouse showed no significant change) (Fig. S9 in the Supplementary material).

To further analyze these differences, mouse spleen cells were fused with P3X to prepare hybridomas to obtain the clones secreting specific antibodies against CH401MAP (Fig. S1B in the Supplementary material). We found that the mean colony number/well was increased in the case of HD-M spleen; however, that of the BC-M spleen was varied following immunization (Table S3 in the Supplementary material). The percentages of wells with CH401MAP-specific IgG were found to be higher in BC-M spleens compared to that of HD-M, although the specific antibody production of almost all of the hybridomas in BC-M spleens was found to be lower than that of HD-M spleen in CH401MAP-immunized mice (Fig. 4B).

These results suggest that cancer-antigen-specific antibody production was observed in BC-M hybridomas, and the clonal deletion of HER2-specific B cells may not have occurred. However, affinity maturation might be blocked in the BC immune system, and the function of antigen-specific IgG accumulation might be disrupted in the immune system of BCs.

### Effects of the anti-PD-1 antibody, nivolumab, on hu-PBL hIL-4 NOG mouse immunity

**Figure 5:**
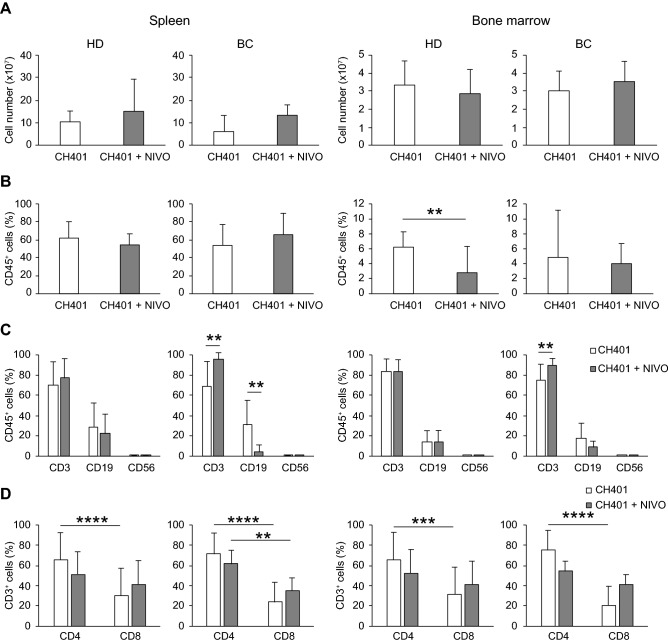
Effects of nivolumab on the cellularity of engrafted human cells. Nivolumab (NIVO) effects on human lymphocytes localized in the spleen and bone marrow were compared between HD-M (CH401MAP; *n* = 17, CH401MAP + NIVOb; *n* = 7) or BC-M (CH401MAP; *n* = 13, CH401MAP + NIVO; *n* = 6). CH401MAP and CH401MAP + NIVO administration shown as white (CH401MAP) or gray (CH401MAP + NIVO) bars. **(A)** Cell number of the spleen and bone marrow. **(B)** Percentage of human CD45^+^ cells. **p = 0.01. **(C)** Percentage of each subset of lymphocytes, CD3^+^ T cells, CD19^+^ B cells, and CD56^+^ natural killer cells. **p = 0.01. **(D)** Percentage of CD4^+^ and CD8^+^ T cells. *p = 0.05, **p = 0.01, ***p = 0.001, ****p = 0.0001. *BC* breast cancer; *HD* healthy donor.

**Figure 6:**
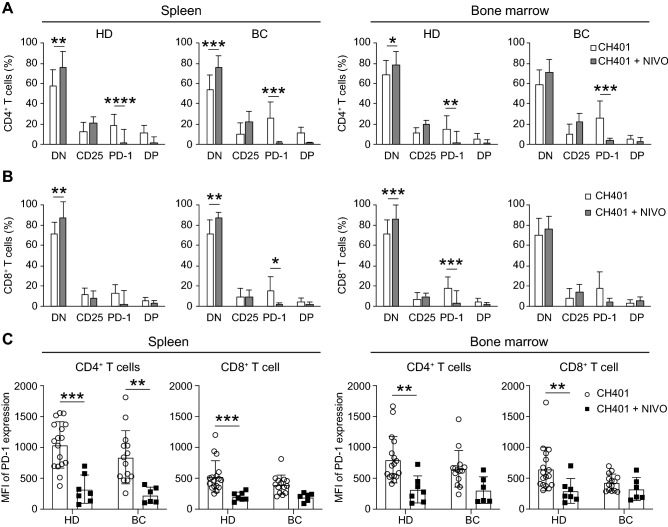
Effects of nivolumab on the activation/exhaustion of engrafted human T cells. Effects of NIVO on human T cells localized in the spleen and bone marrow of mice transplanted with HD (CH401MAP; *n* = 17, CH401MAP + NIVO; *n* = 7) or BC (CH401MAP; *n* = 13, CH401MAP + NIVO; *n* = 6) PBMCs. CD25 and PD-1 expression in **(A)** CD4^+^ T cells and **(B)** CD8^+^ T cells localized in the spleen and bone marrow of mice transplanted with HD or BC PBMCs. Double-negative DN; CD25^−^PD-1^−^, CD25; CD25^+^PD-1^−^, PD-1; CD25^−^PD-1^+^, Double-positive DP; CD25^+^PD-1^+^. CH401MAP and CH401MAP + NIVO administration shown as white (CH401MAP) or gray (CH401MAP + Nivolumab) bars. *p = 0.05, **p = 0.01, ***p = 0.001, ****p = 0.0001. **(C)** MFI of PD-1 expression on CD4^+^ T cells or CD8^+^ T cells in the spleen and bone marrow of hu-PBL hIL-4 NOG mouse. *p = 0.05, **p = 0.01, ***p = 0.001, ****p = 0.0001. *BC* breast cancer; *HD* healthy donor; *PBMCs* peripheral blood mononuclear cells; *MFI* mean fluorescence intensity.

Next, we examined the effects of nivolumab on the humoral immunity of HD-M and BC-M. Initially, we compared the cellularity of CH401MAP-immunized mice and nivolumab-treated, CH401MAP-immunized mice. The typical FCM patterns are displayed in Fig. S10 in the Supplementary Materials. We did not observe any significant decrease in the cell number in both spleen and BM (Fig. 5A), or in the proportion of engrafted CD45^+^ cells in nivolumab-injected spleen cells in HD-M and BC-M. However, there was a decrease in the proportion of CD45^+^ cells in the BM in HD-M (Fig. 5B), suggesting that PD-1 antibodies attacked human lymphocytes to reduce the engrafted ratio in HD-M. In contrast, nivolumab treatment did not induce any changes in the proportions of T cells and B cells of HD-M; however, the proportion of T cells was significantly increased, and CD19^+^ B cell levels were significantly decreased in BC-M (Fig. 5C, CH401 vs. CH401 + NIVO). No significant difference was observed among HER2 subgroups (Fig. S11C in the Supplementary materials). Although the levels of CD8^+^ T cells in HD-M was comparable to those of CD4^+^ T cells, the levels of CD8^+^ T cells remained significantly lower than the levels of CD4^+^ T cells in BC-M (Fig. 5D). No significant difference was observed among HER2 subgroups; however, the HER2 1 + 2 + subset showed a higher ratio of CD8 T cells (Fig. S11D in the Supplementary Materials). In particular, levels of the PD-1^+^ CD4^+^ and CD8^+^ T cells of HD-M and BC-M were downregulated following nivolumab treatment (Fig. 6A,B). No difference was observed between HER2 0, HER2 1 + & 2 + and HER2 3 + subgroups (Fig. S12A,B in the Supplementary materials). In contrast, the proportion of CD25^+^ cells were tended to increase among the HD-M CD4^+^ T cells. A similar tendency was observed in the BC-M spleen. The MFI of PD-1 was significantly downregulated in both CD4^+^ and CD8^+^ T cells of HD-M and BC-M (Fig. 6C). A significant difference was observed only in HER2 1 + 2 + fractions of CD4 T cells (Fig. S12C in the Supplementary materials).

**Figure 7:**
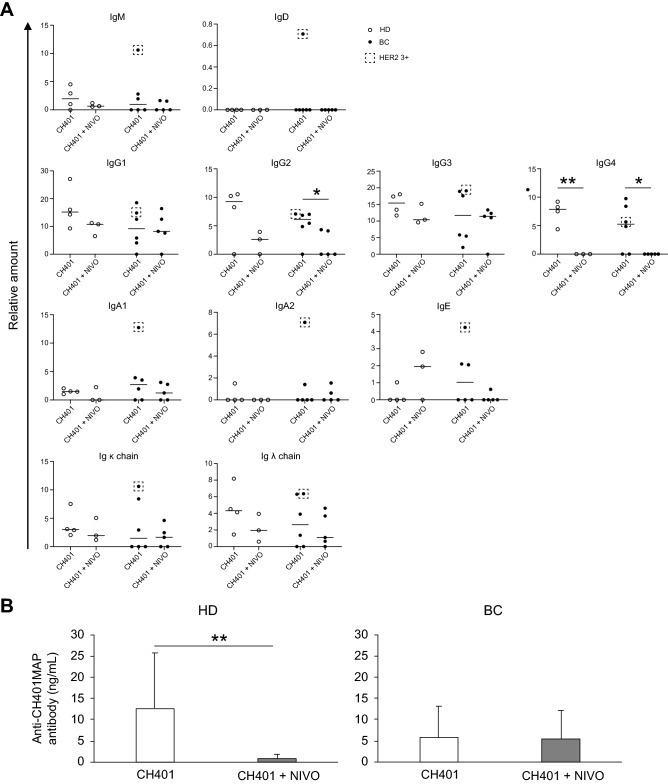
Effects of nivolumab on antibody production by human B cells. **(A)** Relative amount of each human antibody class in HD-transplanted (open circle; CH401MAP; n = 4, CH401MAP + NIVO; n = 3) or BC-transplanted (filled circle; CH401MAP; n = 6, CH401MAP + NIVO; n = 5) mice. HER2 3 + BC-Ms were indicated with dotted squares. *p = 0.05, **p = 0.01. **(B)** Anti-CH401MAP antibody concentrations in HD- or BC-transplanted mouse plasma; CH401MAP (white bar) or CH401MAP + NIVO (gray bar) groups. **p = 0.01. *BC* breast cancer; *HD* healthy donor; *Ig* immunoglobulin.

However, as mentioned above, CD19^+^ B cells were significantly downregulated in nivolumab-treated CH401MAP-immunized BC-M spleen and BM, whereas B cell proportions were not changed following nivolumab injection in HD-M (Fig. 5C). Since most of the B cells found in the mice were plasmablasts, we hypothesized that the antibody production might be suppressed via nivolumab treatment in BC-M. Therefore, we examined plasma Ig levels and CH401MAP-specific antibody production. We found a decrease in almost all of the serum Ig subtypes, and we could not detect IgG4 in the plasma of HD-M and BC-M (Fig. 7A). As shown in the dotted squares, all HER2 3 + samples displayed the highest scores except for IgG1 and IgG4. Similarly, C4a and C4b levels were significantly downregulated in the nivolumab-treated mouse sera (Fig. S7C in the Supplementary material). Nivolumab treatment significantly decreased specific Ab production in HD-M, but not in BC-M, which was originally lower than that observed in HD-M (Fig. 7B). When hybridomas were prepared using these cells, specific-antibody-secreting clones were not detected in HD-M, whereas very-low-specificity-antibody-secreting cells were detected in BC-M (Fig. 4B, Table S3 in the Supplementary material).

Collectively, nivolumab decreased the proportion of exhausted T cells and enhanced CD8^+^ T cell levels and inhibited B cell survival, antibody secretion mediated by B cells, and complement secretion via lymphocytes. As BC lymphocytes were prone to be exhausted, and the cellular and humoral immune systems were disrupted, immune reactivation via nivolumab to enhance cellular immunity and to suppress humoral immunity was attenuated compared to the HD immune system.

## Discussion

In this study, we developed a hu-PBL hIL-4 NOG mouse model that enabled the reconstitution of PB environments in HD and BC patients. Using this mouse model, we simulated the trends in CD4^+^ and CD8^+^ T cell proportions of HDs and BCs in HD-M and BC-M. The HER2 subgroup of BC-M demonstrated high levels of CD25^−^PD-1^+^ exhausted T cells, which exaggerated the patient’s immune cell profile, as reported previously^33^. The results of PCA revealed differences between plasma protein profiles in HD-M and BC-M. Moreover, the humoral immune function that contributed to the production of HER2-specific antibodies was diverse in the BC-M immune system and lowered in the HER2 1 + 2 + (low) group. These results suggest that the humoral immunity of BCs was diverse to block the production of sufficient levels of anti-cancer antibodies in the hu-PBL hIL-4 NOG mice. However, nivolumab administration decreased PD-1-expressing T cell levels in both HD-M and BC-M and the depletion of B cells was predominant in BC-M.

The importance of cytotoxic T cells in cancer immunity is well established. PBMCs derived from BCs are reported to contain significantly lower levels of CD8^+^ T cells compared to those of HD^13^, which shows how the immune system in cancer patients decreases the cytotoxic T cell response. In this study, we determined if the hu-PBL hIL-4 NOG mouse reflected the immune response in BC-M using T cell engraftment as an indicator. Since graft-versus-host disease (GVHD) is typically induced in immunodeficient mice transplanted with human PBMCs, the donor’s immunity might shift to a cytotoxic T cell-dominant type. Nevertheless, the cellularity of HD-M and BC-M was similar to that of the original samples, and CD4^+^ T cells are more abundant in BC-M than in HD-M. This might be because NOG-hIL-4-Tg produced significant amounts of IL-4, which might shift the donor’s immunity to the T helper 2 (Th2)-mediated type. Actually, the immune environment was observed to be shifted slightly towards the Th2-mediated type, as the IgG2 level tended to decrease and IgE started to appear, as shown in Fig. 3B,C. These results suggest the usefulness of the mouse model.

Although the involvement of T cells in cancer immunity has been studied extensively, the role of humoral immunity remains unclear. Most studies use mouse models and a patient’s response against a cancer vaccine or cancer antigen-based antibody in the serum^29,34,35^. In this study, we successfully compared B cell functions in HD and BC using the hu-PBL hIL-4 NOG mouse. Our results showed distinct clusters of HD-M and BC-M, irrespective of CH401MAP immunization. This evidence suggests that the characteristics of humoral immunity are different in HD and BC. The components consisted of human Igs and complement factors. However, previous reports have demonstrated the suppression of cellular immunity and partial enhancement of humoral immunity in tumor-bearing mice^34^. B cell depletion has been shown to effectively delay the development of tumors transplanted into mice^36–38^. In contrast, the Serological analysis of recombinant cDNA expression libraries (SEREX) method was developed for monitoring antitumor antibodies based on the evidence that cancer patients could produce anti-cancer antibodies^39^. In this context, B cell infiltration into a mass of cancer cells is considered a sign of good prognosis^40,41^. Thus, the evidence for the humoral immunity against cancer is controversial. Our findings might shed new light on the role of humoral immunity in cancer.

Complement factors are secreted not only by the liver but also by immune cells^42,43^. Although the hu-PBL hIL-4 NOG mice showed the presence of human lymphoid cells, very few myeloid cells were engrafted and maintained. Therefore, we concluded that the human complement factors detected in these mice were secreted by the engrafted lymphocytes, although production of complement factors in hu-PBL hIL-4 NOG mouse has not been reported thus far. Therefore, this may be a specific phenomenon in NOG-hIL-4-Tg mice. The other detectable human protein molecules comprised cellular proteins, which could be the product of cell lysis. Although PCA showed some differences among the serum proteins, individual factors did not show a high correlation or significant differences. These results suggest that Igs and complement factors could be attributed to the differences between humoral immune systems in HD and BC.

The most remarkable outcome of this study is the phenomenon of varied production of CH401MAP-specific antibody in HER2 subgroups of the immunized BC-M (except HER2 3 +). This suggests that the humoral immune environment in some of BCs, with the exception of HER2 3 + patients, is somewhat dampened compared to the immune environment in HD. Our results are comparable to the findings of previous studies indicating HER2 negative/low cancers induced low-immune-response against anti-HER2 therapy^7–9^. The reason why lymphocytes of two HER2 3 + BC-M could induce anti-HER2 response remains unclear; however, high expression of HER2 on the cancer cells might activate HER2 specific memory B cells and plasma cells, while low expression of HER2 might be insufficient to induce such memory and plasma cells. If the plasma cells are survived in BC-M, it may contribute to the maintenance of specific antibody level in BC-M after nivolumab treatment (Fig. 7B). On the other hand, we observed a significant number of B cell clones producing antibodies with low or no anti-HER2 production in BC-M. Unlike trastuzumab or rituximab treatment, which supply a large amount of high-avidity cancer-specific antibodies, the antibody in this study would not be enough to induce an anti-cancer effect. These results suggest the induction of priming of anti-cancer antibodies in BCs; however, a secondary signal, which induces clonal expansion of specific clones in HDs, might have eliminated highly specific B cells. Therefore, the antibodies identified by Serological analysis of recombinant cDNA expression libraries (SEREX)^39^ might be antibodies with low-specificity and unable to suppress tumor growth. Zahm et al. reported that high-affinity epitopes impaired antitumor efficacy by increasing PD-1 expression on CD8^+^ T cells^44^. A similar phenomenon could occur in BC-M derived from HER2 0 to 2 + patients for CD4^+^ T cells. As Park and collaborators reported that anti-tumor antibodies with high-titer rejected breast cancer of rodent model^45^, B cell function might significantly contribute to reject tumors. Therefore, the malfunction of specific antibody production may be related to the tumor progression.

Moreover, the B cells observed in hu-PBL hIL-4 NOG mice may include regulatory B cells (Bregs), as human Bregs share the same phenotype with plasmablasts (CD27^+^CD28^+^) and may secrete immunosuppressive IL-10, but not high-affinity antibodies^46–48^. As B cell depletion effectively delays the development of tumors transplanted into mice^36–38^, it is possible that Bregs developed in cancer PBMC-bearing mice may suppress T cell function in vivo. Alternatively, B cells might be continuously produced in the patient’s body with no statistically significant changes being detected. Further examination will clarify the detailed characteristics of the engrafted B cells.

Immune checkpoint inhibitors (ICI) were developed to block the interaction of PD-1 molecules on T cells and PD-L1 on antigen-presenting cells with minimal side effects^49^, although these drugs often induce immune adverse events^50^. Owning to the mechanism, T cells can survive and maintain anti-cancer efficacy based on cytotoxicity. These ICIs are powerful tools in cancer treatment^15,16^. However, therapeutic efficacy cannot be easily predicted prior to administration to the patients as efficacy is observed only for ~ 20% of the patients and is not always associated with PD-L1 expression on the tumor tissue^51,52^. In this study, we observed an increase in CD8^+^ T cell levels in the HD-M spleen along with enhanced CD25 expression following nivolumab treatment. This evidence was consistent with the findings of previous studies on human patients as ICIs have been shown to enhance CD8^+^ T cell infiltration^53–55^. However, in our results, the administration of nivolumab significantly decreased the expression of PD-1 on T cells. This is contrary to the widely accepted hypothesis that PD-1-expressing cells survive following nivolumab administration and kill cancer cells. Because PD-1 pathway was reported to regulate development and function of memory CD8 T cells^56^, it might affect the regulation of memory CD4 T cells in a similar manner. In our system, most of the engrafted cells are memory phenotype. Moreover, the immunization schedule is based on the memory formation. Therefore, PD-1 blockage might inhibit the memory T cell development in the mice. The effector T cells that survived to PD-1 blockage might have rather short lifespans and not be detected after 4 weeks which may affect the secretion of antibodies and complements. On the other hand, most of the BC-Ms which significantly decreased the PD-1 expression on CD4 T cells after nivolumab treatment are HER2 1 + & 2 + subgroups (Fig. S12). Further analysis comparing the BC types might clarify the molecular mechanism by which the nivolumab-treated mice decrease PD-1 positive T cells.

PD-1 is expressed on T follicular helper (Tfh) cells and plays a pivotal role in germinal center reactions to produce high-titers of antigen-specific antibodies^57^. However, the benefits of PD-1/PD-L1 signals have not been fully understood yet. A subset of B cells with low levels of antigen specificity have recently been reported to express PD-L1 and increase the threshold of antigen-specific antibody production in B cells^58^. This is consistent with our results as we observed low-affinity clones expressing low levels of PD-L1 on plasmablasts. On the other hand, plasmablasts in BC-M disappeared following nivolumab treatment. Since the expression levels of PD-1 were very low in B cells (Fig. S6 in the Supplementary material), we speculated that the PD-1/PD-L1 interaction between Th cells and B cells expressing low levels of PD-L1 is crucial for B cell survival.

In conclusion, we have shown that immune systems in HD and BC are different and that the humoral immunity of BC was diverse in the hu-PBL hIL-4 NOG mice. The hu-PBL hIL-4 NOG mouse used in this study is a promising tool to analyze the patient humoral immune systems and could be utilized in the practice of personalized medicine or the development of new immune-related treatment.

## Methods

### Ethical approval

Human PBMCs were derived from HDs and BCs after receiving written informed consent following protocol approval by the Institutional Review Board according to the institutional guidelines. This work was approved by the Tokai University Human Research Committee (12R-002) and the Central Institute for Experimental Animals (CIEA) Human Research Committee (08-01). These studies were conducted in accordance with the guidelines of the Declaration of Helsinki and the Japanese federal regulations required for the protection of human subjects. Immunodeficient mice were used for xenotransplantation studies in compliance with the Guidelines for the Care and Use of Laboratory Animals, and all animal protocols were approved by the committees of CIEA (#20045) and the Tokai University School of Medicine (#185016, #191073, #202049). This study was carried out in compliance with the ARRIVE guidelines.

### Patients and healthy donors

Histologically confirmed cases of BC, with no history of other malignant diseases, who had not yet undergone surgery admitted to the Tokai University Hospital in Kanagawa, Japan from September 2014 to January 2017 were recruited for this study. The expression level scores of HER2, estrogen receptor, progesterone receptor, and Ki-67 were estimated in the main tumor by immunohistochemistry. The diagnosis from HER2 0 to HER2 3 + was performed according to the ASCO/CAP2013 Guideline with the confirmation of the co-author's Board-Certified Fellow of the Japan Breast Cancer Society. In HER2 2 + cases, a dual-color in situ hybridization (DISH) test was performed. Blood derived from healthy donors without any history of malignant diseases were selected as controls. Table 1 summarizes the detailed patient information. HDs (n = 39, range 20‒68 year-old) and BCs were enrolled in the study for PBMC analysis (n = 47, range 35–87 year-old) (Table 1A) and for the development of hu-PBL hIL-4 NOG mouse (n = 16, range 35‒82 year-old) (Table 1B) were enrolled in the study.

**Table 1:** Characteristics of the enrolled breast cancer patients.

	Age	Diagnosis	Tumor size (mm × mm)	Lymph node metastasis	Subtype	ER	PgR	HER2	Ki67	Analyzed in
**A**										
B-A3	35	Papillotubular carcinoma	38 × 20	0	Luminal B	80	10	1	20	Figure 1A
B-A4	64	Noninvasive ductal carcinoma	–	0	HER2 type	0	0	3	40	
B-A5	48	Noninvasive ductal carcinoma	–	0	Luminal B	90	0	3	10	
B-A6	49	Noninvasive ductal carcinoma	–	0	HER2 type	0	0	2, DISH(−)	20	
B1	65	Scirrhous carcinoma	30 × 25	0	Luminal B	90	0	1	20	
B2	59	Mucinous carcinoma	25 × 15	0	Luminal A	90	30	0	10	
B3	50	Papillotubular carcinoma	12 × 12	0	Luminal A	90	90	1	5	
B5	56	Papillotubular carcinoma	10 × 10	0	Triple negative	0	0	1	10	
B6	57	Scirrhous carcinoma	28 × 14	0	Triple negative	0	0	0	50	
B7	71	Papillotubular carcinoma	20 × 15	1	Triple negative	0	0	0	20	
B8	67	Papillotubular carcinoma	15 × 15	0	Luminal A	90	80	1	5	
B9	62	Scirrhous carcinoma	15 × 10	0	Luminal B	90	0	1	10	
B10	58	Scirrhous carcinoma	30 × 25	0	Luminal B	100	5	0	10	
B11	73	Mucinous carcinoma	65 × 70	1	Luminal A	90	60	1	10	
B12	72	Mucinous carcinoma	17 × 10	0	Triple negative	0	0	0	20	
B13	67	Mucinous carcinoma	22 × 10	0	Triple negative	0	0	1	5	
B14	51	Papillotubular carcinoma	10 × 10	0	Triple negative	0	0	0	5	
B15	48	Papillotubular carcinoma	16 × 15	0	Luminal A	70	60	1	5	
B16	65	Papillotubular carcinoma	61 × 59	0	Luminal A	90	70	1	10	
B17	48	Papillotubular carcinoma	15 × 5	0	Luminal A	10	5	0	5	
B18	39	Scirrhous carcinoma	60 × 23	1	Luminal B	60	10	3	30	
B19	60	Papillotubular carcinoma	90 × 60	0	Triple negative	0	0	0	20	
B20	74	Papillotubular carcinoma	90 × 80	1	Luminal B	5	0	1	20	
B21	73	Mucinous carcinoma	15 × 10	0	Luminal A	90	80	0	20	
B22	46	Noninvasive ductal carcinoma	–	0	Luminal B	70	70	3	5	
B24	49	Papillotubular carcinoma	16 × 12	0	Luminal B	70	70	2, DISH(−)	5	
B27	50	Mucinous carcinoma	20 × 10	0	Luminal A	90	40	1	10	
B29	50	Mucinous carcinoma	20 × 10	0	Luminal A	90	40	1	10	
B30	69	Apocrine carcinoma	16 × 16	0	HER2 type	0	0	2, DISH(−)	20	
B31	63	Invasive lobular carcinoma	14 × 5	0	Luminal B	90	90	2, DISH(−)	5	
B32	39	Papillotubular carcinoma	45 × 43	1	HER2 type	0	0	3	70	
B33	87	Papillotubular carcinoma	17 × 16	0	Luminal B	90	0	1	20	
B34	82	Mucinous carcinoma	110 × 85	1	Luminal B	90	10	2, DISH(−)	10	
B35	61	Invasive lobular carcinoma	32 × 27	0	Luminal A	80	60	0	5	
B36	59	Papillotubular carcinoma	25 × 20	1	Luminal A	90	90	1	20	
B37	62	Papillotubular carcinoma	50 × 40	0	Luminal B	90	0	2, DISH(−)	10	
B39	64	Mucinous carcinoma	165 × 155	1	Luminal B	90	0.02	1	10	
B40	78	Solid-tubular carcinoma	22 × 14	0	Triple negative	0	0	0	70	
B41	50	Papillotubular carcinoma	12 × 9	0	Luminal A	70	0.9	1	10	
B42	44	Papillotubular carcinoma	45 × 6	0	Luminal A	50	0.7	1	10	
B43	42	Scirrhous carcinoma	15 × 15	0	Luminal A	90	0.9	0	5	
B44	51	Papillotubular carcinoma	25 × 15	1	Luminal A	80	0.8	1	10	
B45	72	Papillotubular carcinoma	16 × 15	0	HER2 type	0	0	3	30	
B47	82	Invasive micropapillary carcinoma (Left)	115 × 55	1	Triple negative	0	0	1	20	
		Invasive ductal carcinoma (Right)	20 × 20	1	Luminal A	90	0	0	10	
B48	70	Tubular forming carcinoma	28 × 15	0	Luminal-HER2	90	0	3	5	
B49	47	Tubular forming carcinoma	7 × 6	0	Luminal A	90	90	2, DISH(−)	10	
B52	43	Invasive ductal carcinoma	44 × 35	1	Luminal B	90	90	2, DISH(−)	40	
**B**										
B-A3	35	Papillotubular carcinoma	38 × 20	0	Luminal B	80	10	1	20	Figure 1B
B34	82	Mucinous carcinoma	110 × 85	1	Luminal B	90	10	2, DISH(−)	10	Figure 1B, 2, 3, 4, 5, 6, 7
B35	61	Invasive lobular carcinoma	32 × 27	0	Luminal A	80	60	0	5	Figure 1B, 2, 4, 5, 6
B36	59	Papillotubular carcinoma	25 × 20	1	Luminal A	90	90	1	20	Figure 1B, 2, 4, 5, 6
B37	62	Papillotubular carcinoma	50 × 40	0	Luminal B	90	0	2, DISH(−)	10	Figure 1B, 2, 3, 4, 5, 6, 7
B38	79	Scirrhous carcinoma	21 × 17	1	Triple negative	0	0	0	40	Figures 5, 6
B39	64	Mucinous carcinoma	165 × 155	1	Luminal B	90	0.02	1	10	Figures 1B, 2, 4, 5, 6
B40	78	Solid-tubular carcinoma	22 × 14	0	Triple negative	0	0	0	70	Figures 1B, 2, 4, 5, 6
B41	50	Papillotubular carcinoma	12 × 9	0	Luminal A	70	0.9	1	10	Figures 1B, 2, 3, 4, 5, 6, 7
B42	44	Papillotubular carcinoma	45 × 6	0	Luminal A	50	0.7	1	10	Figures 1B, 2, 4, 5, 6
B43	42	Scirrhous carcinoma	15 × 15	0	Luminal A	90	0.9	0	5	Figures 1B, 2, 4, 5, 6
B44	51	Papillotubular carcinoma	25 × 15	1	Luminal A	80	0.8	1	10	Figure 2, 3, 4, 5, 6, 7
B45	72	Papillotubular carcinoma	16 × 15	0	HER2 type	0	0	3	30	Figures 2, 3, 4, 5, 6, 7
B46	45	Invasive ductal carcinoma	4 × 4	0	Luminal A	60	20	2, DISH(−)	10	Figures 3, 7
B48	70	Invasive ductal carcinoma	28 × 15	0	Luminal-HER2	90	0	3	5	Figures 4, 5, 6
B54	47	Invasive ductal carcinoma	63 × 26	1/3	Luminal B	30	0	1	20	Figure 1B

*ER* estrogen receptor; *PgR* progesterone receptor; *HER2* Human epidermal growth factor receptor 2; *DISH* dual-color in situ hybridization.

### NOG-hIL-4-Tg

NOD/Shi-scid-IL2rγ^null^-hIL-4 Tg (NOG; formal name, NOD.Cg-Prkdc^scid^Il2rg^tm1Sug^/ShiJic) mice were maintained in Tokai University School of Medicine under specific pathogen-free conditions. Offsprings with the expression of inserted transgene were identified as described previously^12^. The NOG-hIL-4-Tg mice were housed under specific pathogen-free conditions in the animal facility located at CIEA or Tokai University School of Medicine during the experiments. DNA was extracted from the ear tissues collected at the time of genotyping.

### Preparation and transplantation of human PBMCs

A total of 7.5 mL of PB from each HD or BC was collected in the morning of their surgery using Vacutainer ACD tubes (NIPRO Corporation, Japan, Osaka) containing heparin. The collected PB was immediately placed in 10 mL Ficoll-Hypaque (SIGMA-ALDRICH, London, UK), and mononuclear cells were isolated by density centrifugation (500×*g*, 30 min, 20 °C). The cells were washed with phosphate-buffered saline (PBS) for 5 min at 300×*g* at 4 °C. Around 2.5 to 5 × 10^6^ PBMCs were transplanted intravenously into 8‒12-week-old NOG or NOG-hIL-4-Tg mice. The number of mice transplanted depended on the amount of available material, in particular the BC PBMCs. The mice transplanted with HD PBMCs were referred to as HD-M, and those transplanted with BC PBMCs as BC-M.

### Analysis of engrafted human cells in hu-PBL hIL-4 NOG mice

Four weeks post transplantation, the mice were euthanized and heparinized PB was collected via retro-orbital bleeding. The mice were sacrificed and analyzed 28 days after transplantation. The engraftment and the cellularity of human cells in the bone marrow (BM), spleen, and PB were analyzed via flow cytometry as described below. Mononuclear cells were counted, and the reconstitution rate of human cells was determined based on hCD45 expression in the lymphoid-gated fraction.

The 5 × 10^5^ cells were stained with 1 μL of appropriately diluted fluorescently labeled monoclonal antibodies (mAbs) for 15 min at 4 °C and analyzed using FACS Fortessa (BD Bioscience, Franklin Lakes, NJ, USA). For each analysis, the live white blood cells or lymphocytes were further gated based on hCD45 expression. The mAbs and the dilution factors are summarized in Table S1 in the Supplementary material.

### Peptide immunization

CH401 multiple antigen peptide (MAP), which contains the epitope sequence of the anti-HER2 mAb CH401, was used as the cancer antigen^31^. The CH401MAP peptide was synthesized using a Rink amide resin (0.4‒0.7 mmol g^−1^), An ACT357 peptide synthesizer (Advanced Chemtech, Louisville, KY, USA). BAL6MAP, which contains a partial amino acid sequence of *Pseudomonas aeruginosa* BAM A (NYYAGGFNSVRGFKDSTLGP), was used as the control peptide^59^. CH401MAP was emulsified with complete Freund’s Adjuvant (Wako Pure Chemical Industries, Ltd, Osaka, Japan) (50 μg/head, 100 μL 1:1 v/v) and administered to the hu-PBL hIL-4 NOG mice intraperitoneally. For the negative control, an equal volume of PBS was emulsified and injected into hu-PBL hIL-4 NOG mice. Boosters were administered using Freund’s incomplete adjuvant (Wako Pure Chemical Industries, Ltd) 2 weeks after the first immunization. Two weeks after the booster, the mice were sacrificed and used for subsequent analysis.

### Enzyme-linked immuno-sorbent assay (ELISA)

Human IL-4 protein levels were measured using the Human IL-4 ELISA Set BD OptEIA™ Kit (BD Biosciences) according to the manufacturer’s instructions. The protocol for specific IgG antibody detection has been previously described^12^. Briefly, micro-wells of microtiter plates (Thermo Fisher Scientific, MA, USA) were coated overnight with MAPs (2 μg mL^−1^) diluted in carbonate buffer (pH 9.5) at 4 °C. The wells were washed with PBS-Tween (0.05% v/v) and blocked with 3% BSA-PBS at room temperature (RT) for 1 h. After the washes, serial dilutions of mouse plasma were added and incubated at RT for 2 h. The plates were washed and biotin-conjugated mouse anti-human IgG mAb (Bethyl Laboratories, TX, USA) (1:1000) was added. After a 1 h incubation at RT, the plates were washed, followed by the addition of streptavidin–horseradish peroxidase (1:1000 v/v; Bethyl Laboratories) and incubated at RT for 1 h. Unbound conjugates were removed through washing. EIA substrate kit solution (Bio-Rad Laboratories, Hercules, CA, USA) was added to each well. The reaction was stopped using 10% H_2_SO_4_, and the absorbance was measured at 450 nm. For the quantification of specific antibodies, the wells were coated with anti-human Igs. A known concentration of the purified human IgG was prepared and added as the antigen mimicking human anti-CH401MAP IgG, and based on the absorbance, a standard curve was plotted. The concentration of the specific IgG in the serum was then calculated from the standard curve. CH401MAP-positive and BAL6MAP-negative clones were counted as CH401MAP-specific wells.

### Liquid chromatography-ion trap mass spectrometry (LC–MS/MS) analysis

LC–MS/MS was performed as described previously^60,61^. Briefly, plasma (2 µL) was diluted with 50 mM Triethylammonium bicarbonate and digested with 200 ng µL^−1^ trypsin solution at 37 °C for 20 h. Then, 100 mM dithiothreitol was added to the lysates and incubated at 95 °C for 5 min, and dried using a Speed Vac (Thermo Scientific, Inc., Waltham, MA, USA). For LC–MS/MS analysis, the lysates were dissolved in 0.1% trifluoroacetic acid and injected into an UltiMate 3000 HPLC system (Thermo Scientific, Inc.). A reverse-phase column (Develosil 300ODS-HG-5; 1.0 mm internal diameter × 100 mm; Nomura Chemical Co. Ltd., Seto, Japan) was used at a flow rate of 50 µL min^−1^ with a 0 − 60% linear gradient of acetonitrile in 0.1% formic acid. Eluted peptides were subjected to QExactive hybrid quadrupole-Orbitrap mass spectrometry (Thermo Scientific Inc.). The mass acquisition method consisted of one full MS survey scan followed by an MS/MS scan of the most abundant precursor ions from the survey scan. Data were analyzed using Proteome Discoverer (Thermo Scientific Inc.), Mascot (Matrix Science Inc., Boston, MA, USA), and Scaffold (Proteome Software Inc., Portland, OR, USA). The NCBI database (GenBank) was used (https://www.ncbi.nlm.nih.gov/), and tandem mass spectra (MS/MS) were searched against the SwissProt database (https://www.uniprot.org/). The Scaffold software was used for label-free quantitation. Using a target decoy approach, a peptide false discovery rate of 0.2% was determined. Comparisons between each sample were made by normalizing the spectrum counting values of all identified proteins using the Scaffold software. The relative amount of Ig concentration was obtained by normalizing the spectrum counting values.

### Hybridoma preparation and characterization of human B cells engrafted in the spleen of the hu-PBL hIL-4 NOG mouse

The hu-PBL hIL-4 NOG mouse splenocytes were fused with the mouse myeloma cell line, P3-X63-Ag8-U1, using electroporation with a BEX CFB16-HB (BEX co. LTD, Tokyo, JPN) and the electrode, LF497P2. The conditions for electroporation were set at AC 30 V for 20 s, DC 350 V for 30 µs/500 ms DC cycle 3, AC 30 V for 7 s, Fade on, in electrofusion buffer (0.3 M mannitol, 0.1 mM calcium chloride, and 0.1 mM magnesium chloride). Fused cells were cultured in HAT medium for two weeks; LC–MS/MS and ELISA were performed using the supernatants for the measurement of total Ig and CH401MAP-specific IgG levels, as described previously.

### Statistics

Statistical analysis was performed using Microsoft Excel (Microsoft, Redmond, WA, USA) or GraphPad Prism 8 (GraphPad, San Diego, CA, USA). The data are represented as the mean ± Standard Deviation. Significant differences between groups were determined using two-sided Student’s *t*-test or One-way ANOVA. SIMCA 13.0 (Sartorius Stedim Biotech, Goettingen, Germany) was used for principal component analysis (PCA).

## Supplementary Information

Supplementary Information.

## Data Availability

The datasets generated for this study are available on request to the corresponding author.

## References

[1] O'Mahony D, Bishop M (2006). Monoclonal antibody therapy. Front. Biosci..

[2] Maximiano S, Magalhães P, Guerreiro M, Morgado M (2016). Trastuzumab in the treatment of breast cancer. BioDrugs.

[3] Marcus R, Hagenbeek A (2007). The therapeutic use of rituximab in non-Hodgkin's lymphoma. Eur. J. Haematol. Suppl..

[4] Mittendorf E (2016). Primary analysis of a prospective, randomized, single-blinded phase II trial evaluating the HER2 peptide GP2 vaccine in breast cancer patients to prevent recurrence. Oncotarget.

[5] Schwartzentruber D (2011). gp100 peptide vaccine and interleukin-2 in patients with advanced melanoma. N Engl J Med.

[6] Yamada A, Sasada T, Noguchi M, Itoh K (2013). Next-generation peptide vaccines for advanced cancer. Cancer Sci..

[7] Loi S (2013). Prognostic and predictive value of tumor-infiltrating lymphocytes in a phase III randomized adjuvant breast cancer trial in node-positive breast cancer comparing the addition of docetaxel to doxorubicin with doxorubicin-based chemotherapy: BIG 02–98. J. Clin. Oncol..

[8] Stanton S, Adams S, Disis M (2016). Variation in the incidence and magnitude of tumor-infiltrating lymphocytes in breast cancer subtypes: A systematic review. JAMA Oncol..

[9] Luen S, Savas P, Fox S, Salgado R, Loi S (2017). Tumour-infiltrating lymphocytes and the emerging role of immunotherapy in breast cancer. Pathology.

[10] Qin Z (1998). B cells inhibit induction of T cell-dependent tumor immunity. Nat. Med..

[11] Gentles A (2015). The prognostic landscape of genes and infiltrating immune cells across human cancers. Nat. Med..

[12] Kametani Y (2017). NOG-hIL-4-Tg, a new humanized mouse model for producing tumor antigen-specific IgG antibody by peptide vaccination. PLoS ONE.

[13] Falci C (2013). Immune senescence and cancer in elderly patients: Results from an exploratory study. Exp. Gerontol..

[14] Tsuda B (2017). B-cell populations are expanded in breast cancer patients compared with healthy controls. Breast Cancer.

[15] Tumeh P (2014). PD-1 blockade induces responses by inhibiting adaptive immune resistance. Nature.

[16] Sharma P, Hu-Lieskovan S, Wargo J, Ribas A (2017). Primary, adaptive, and acquired resistance to cancer immunotherapy. Cell.

[17] Robert C (2020). Immunotherapy discontinuation: How, and when? Data from melanoma as a paradigm. Nat. Rev Clin. Oncol..

[18] Topalian S, Taube J, Anders R, Pardoll D (2016). Mechanism-driven biomarkers to guide immune checkpoint blockade in cancer therapy. Nat. Rev. Cancer.

[19] Sun C, Mezzadra R, Schumacher T (2018). Regulation and function of the PD-L1 checkpoint. Immunity.

[20] Zuazo M (2017). Molecular mechanisms of programmed cell death-1 dependent T cell suppression: relevance for immunotherapy. Ann. Transl. Med..

[21] Brown J (2003). Blockade of programmed death-1 ligands on dendritic cells enhances T cell activation and cytokine production. J. Immunol..

[22] Balar A, Weber J (2017). PD-1 and PD-L1 antibodies in cancer: Current status and future directions. Cancer Immunol. Immunother..

[23] Yokosuka T (2012). Programmed cell death 1 forms negative costimulatory microclusters that directly inhibit T cell receptor signaling by recruiting phosphatase SHP2. J. Exp. Med..

[24] Shlomchik MJ, Weisel F (2012). Germinal center selection and the development of memory B and plasma cells. Immunol. Rev.

[25] Pardoll D (2012). The blockade of immune checkpoints in cancer immunotherapy. Nat. Rev. Cancer.

[26] Jiang X (2019). Role of the tumor microenvironment in PD-L1/PD-1-mediated tumor immune escape. Mol. Cancer.

[27] Shultz LD (2000). NOD/LtSz-Rag1null mice: An immunodeficient and radioresistant model for engraftment of human hematolymphoid cells, HIV infection, and adoptive transfer of NOD mouse diabetogenic T cells. J. Immunol..

[28] Ito M (2002). NOD/SCID/gamma(c)(null) mouse: An excellent recipient mouse model for engagement of human cells. Blood.

[29] Kametani Y (2019). Humanized mice as an effective evaluation system for peptide vaccines and immune checkpoint inhibitors. Int. J. Mol. Sci..

[30] Seki T (2018). Expression of glucocorticoid receptor shows negative correlation with human B-cell engraftment in PBMC-transplanted NOGhIL-4-Tg mice. Biosci. Trends.

[31] Miyako H (2011). Antitumor effect of new HER2 peptide vaccination based on B cell epitope. Anticancer Res..

[32] Ito R (2009). Highly sensitive model for xenogenic GVHD using severe immunodeficient NOG mice. Transplantation.

[33] Formenti S (2019). Baseline T cell dysfunction by single cell network profiling in metastatic breast cancer patients. J. Immunother. Cancer.

[34] Tan T-T, Coussens L (2007). Humoral immunity, inflammation and cancer. Curr. Opin. Immunol..

[35] Berzofsky J (2017). Cancer vaccine strategies: Translation from mice to human clinical trials. Cancer Immunol. Immunother..

[36] Brodt P, Gordon J (1978). Anti-tumor immunity in B lymphocyte-deprived mice, I. Immunity to a chemically induced tumor. J. Immunol..

[37] Barbera-Guillem E (2000). B lymphocyte pathology in human colorectal cancer. Experimental and clinical therapeutic effects of partial B cell depletion. Cancer Immunol. Immunother..

[38] Monach P, Schreiber H, Rowley D (1993). CD4+ and B lymphocytes in transplantation immunity. II. Augmented rejection of tumor allografts by mice lacking B cells. Transplantation.

[39] Jäger D, Taverna C, Zippelius A, Knuth A (2004). Identification of tumor antigens as potential target antigens for immunotherapy by serological expression cloning. Cancer Immunol. Immunother..

[40] Ladányi A (2015). Prognostic and predictive significance of immune cells infiltrating cutaneous melanoma. Pigment Cell Melanoma Res..

[41] Ladányi A (2011). Prognostic impact of B-cell density in cutaneous melanoma. Cancer Immunol. Immunother..

[42] Lubbers R, van Essen M, van Kooten C, Trouw L (2017). Production of complement components by cells of the immune system. Clin. Exp. Immunol..

[43] West E, Kolev M, Kemper C (2018). Complement and the regulation of T cell responses. Annu. Rev. Immunol..

[44] Zahm C, Colluru V, McNeel D (2017). Vaccination with high-affinity epitopes impairs antitumor efficacy by increasing PD-1 expression on CD8 + T cells. Cancer Immunol. Res..

[45] Park J (2008). Therapy of advanced established murine breast cancer with a recombinant adenoviral ErbB-2/neu vaccine. Cancer Res..

[46] Bouaziz J, Yanaba K, Tedder T (2008). Regulatory B cells as inhibitors of immune responses and inflammation. Immunol. Rev..

[47] Yanaba K (2008). A regulatory B cell subset with a unique CD1dhiCD5+ phenotype controls T cell-dependent inflammatory responses. Immunity.

[48] Zhou X, Su Y, Lao X, Liang Y, Liao G (2016). CD19(+)IL-10(+) regulatory B cells affect survival of tongue squamous cell carcinoma patients and induce resting CD4(+) T cells to CD4(+)Foxp3(+) regulatory T cells. Oral Oncol..

[49] Nishijima T, Shachar S, Nyrop K, Muss H (2017). Safety and tolerability of PD-1/PD-L1 inhibitors compared with chemotherapy in patients with advanced cancer: A meta-analysis. Oncologist..

[50] González-Rodríguez E, Rodríguez-Abreu D (2016). Immune checkpoint inhibitors: Review and management of endocrine adverse events. Oncologist.

[51] Shen X, Zhao B (2018). Efficacy of PD-1 or PD-L1 inhibitors and PD-L1 expression status in cancer: Meta-analysis. BMJ.

[52] Alsaab H (2017). PD-1 and PD-L1 checkpoint signaling inhibition for cancer immunotherapy: Mechanism, combinations, and clinical outcome. Front. Pharmacol..

[53] Peske J, Woods A, Engelhard V (2015). Control of CD8 T-cell infiltration into tumors by vasculature and microenvironment. Adv. Cancer Res..

[54] Wallin J (2016). Atezolizumab in combination with bevacizumab enhances antigen-specific T-cell migration in metastatic renal cell carcinoma. Nat. Commun..

[55] Vilain R (2017). Dynamic changes in PD-L1 expression and immune infiltrates early during treatment predict response to PD-1 blockade in melanoma. Clin. Cancer Res..

[56] Pauken K (2020). The PD-1 pathway regulates development and function of memory CD8 + T cells following respiratory viral infection. Cell. Rep..

[57] Song W, Craft J (2019). T follicular helper cell heterogeneity: Time, space, and function. Immunol. Rev..

[58] Shi J (2018). PD-1 controls follicular T helper cell positioning and function. Immunity.

[59] Gu Y (2016). Structural basis of outer membrane protein insertion by the BAM complex. Nature.

[60] Kametani F (2016). Mass spectrometric analysis of accumulated TDP-43 in amyotrophic lateral sclerosis brains. Sci. Rep..

[61] Kashiwagi H (2020). Human PZP and common marmoset A2ML1 as pregnancy related proteins. Sci. Rep..

