# Supplementary material for: HER2-antigen-specific humoral immune response in breast cancer lymphocytes transplanted in hu-PBL hIL-4 NOG mice
Source: Sci Rep. 2021 Jun 17;11:12798. doi: 10.1038/s41598-021-92311-y (PMC8211648; doi:10.1038/s41598-021-92311-y)
Supplement: Supplementary file 1 — Supplementary Information. [file 41598_2021_92311_MOESM1_ESM.pdf]

Supplementary Information

HER2-antigen-specific humoral immune response in breast cancer lymphocytes transplanted in hu-PBL hIL-4 NOG mice

Yusuke Ohno, Shino Ohshima, Asuka Miyamoto, Fuyuki Kametani, Ryoji Ito, Banri Tsuda, Yukie Kasama, Shunsuke Nakada, Hirofumi Kashiwagi, Toshiro Seki, Atsushi Yasuda, Kiyoshi Ando, Mamoru Ito, Yutaka Tokuda, Yoshie Kametani*

Figure S1

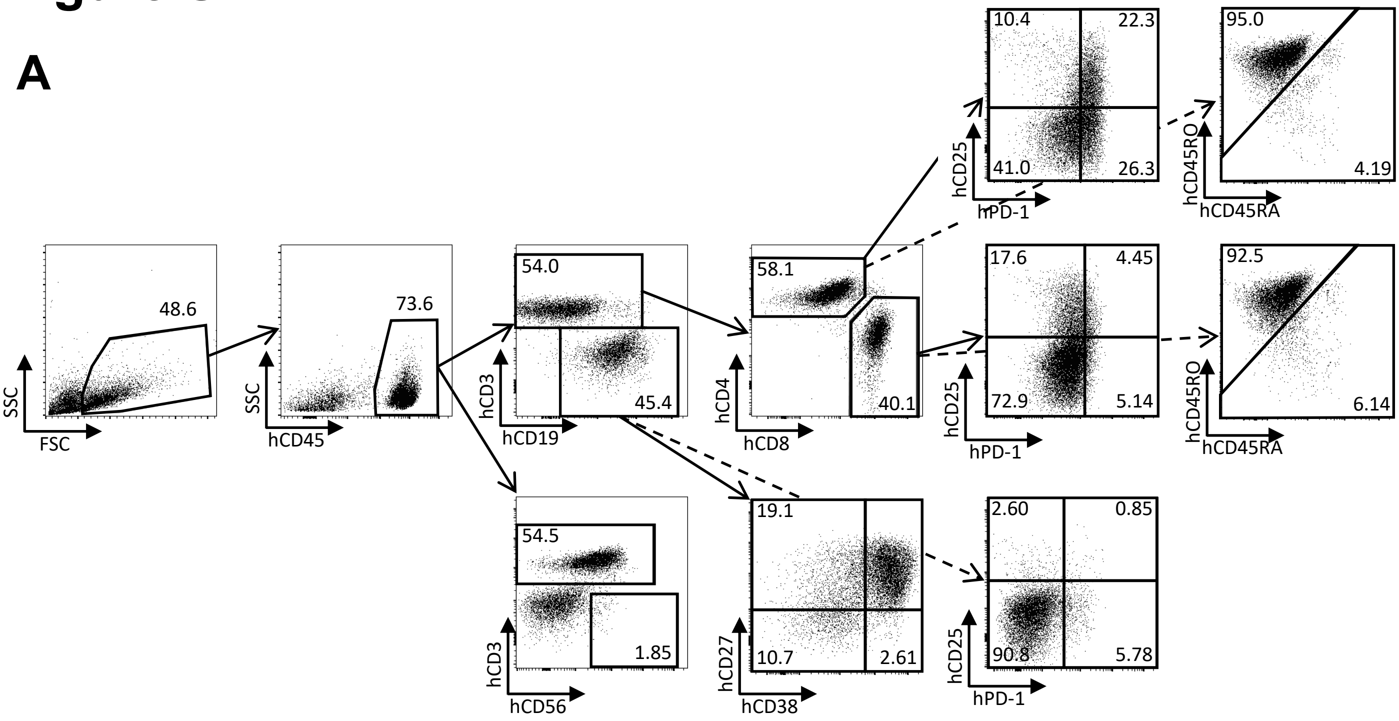

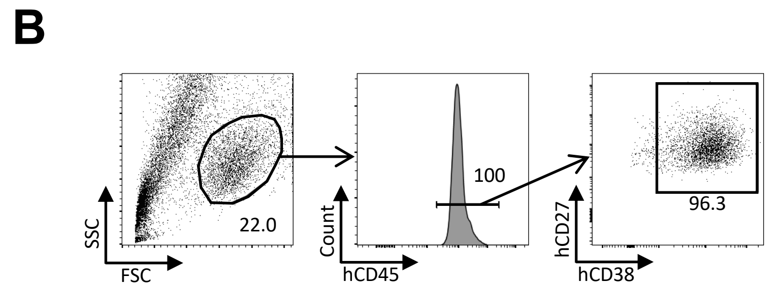

Figure S1 Typical patterns and gating of human lymphocytes in FCM.
Arrows show the fractions analyzed in the next gate, and the percentage of the gated cells were shown in the panels. **(A)** Typical patterns of each human lymphocyte subset and their activation/exhaustion level. **(B)** Typical expression pattern of human plasmablast markers on the hybridoma.

Figure S2

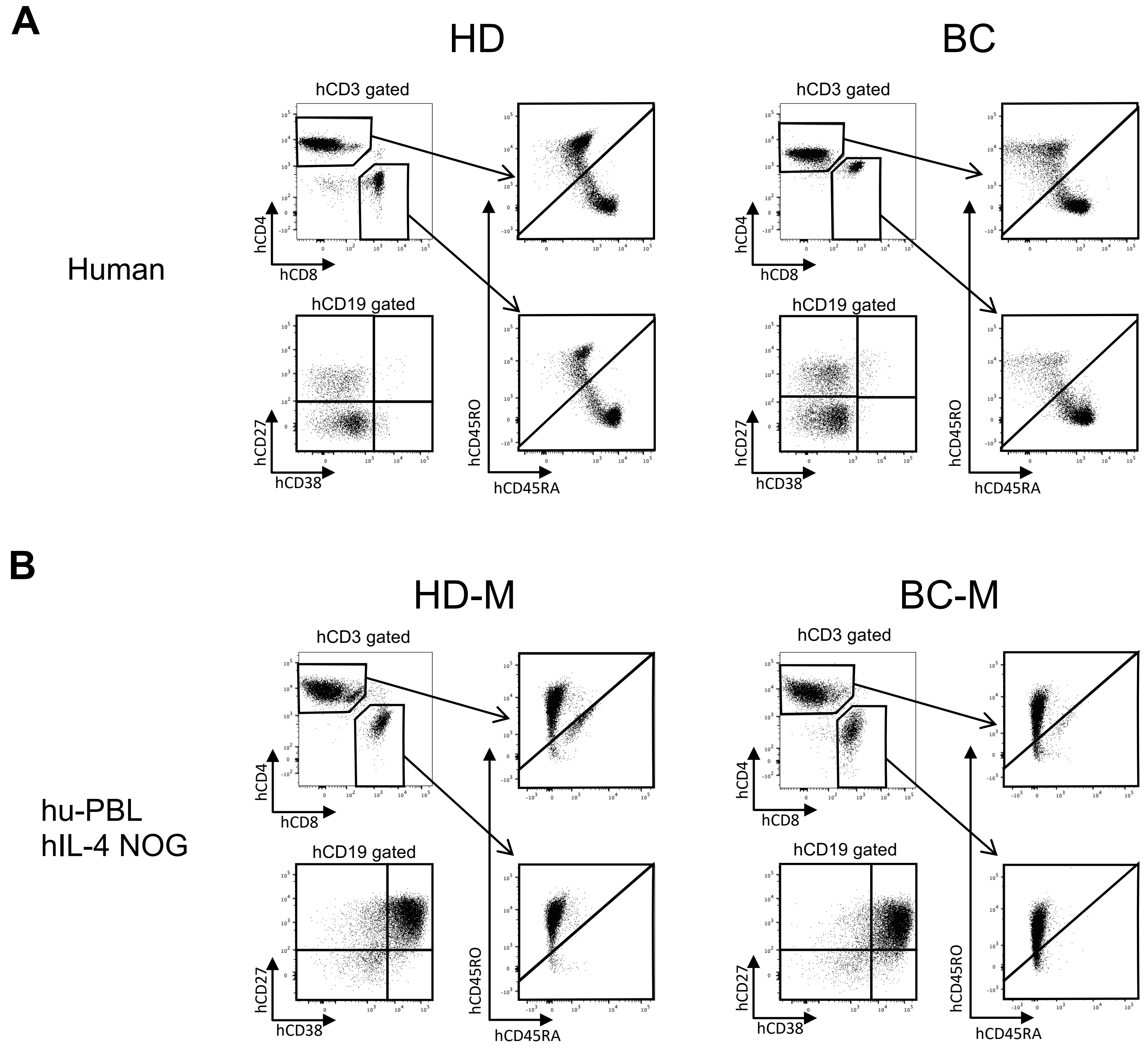

Figure S2 Typical patterns and gating of human T cells and B cells in FCM used in Fig. 1. Arrows show the fractions analyzed in the next gate. **(A)** Typical patterns of memory T cells and plasmablasts in human PBMC of HD or BC. **(B)** Typical patterns of memory T cells and plasmablasts in hu-PBL hIL-4 NOG spleens transplanted with HD or BC PBMCs. BC, breast cancer; HD, healthy donor.

Figure S3

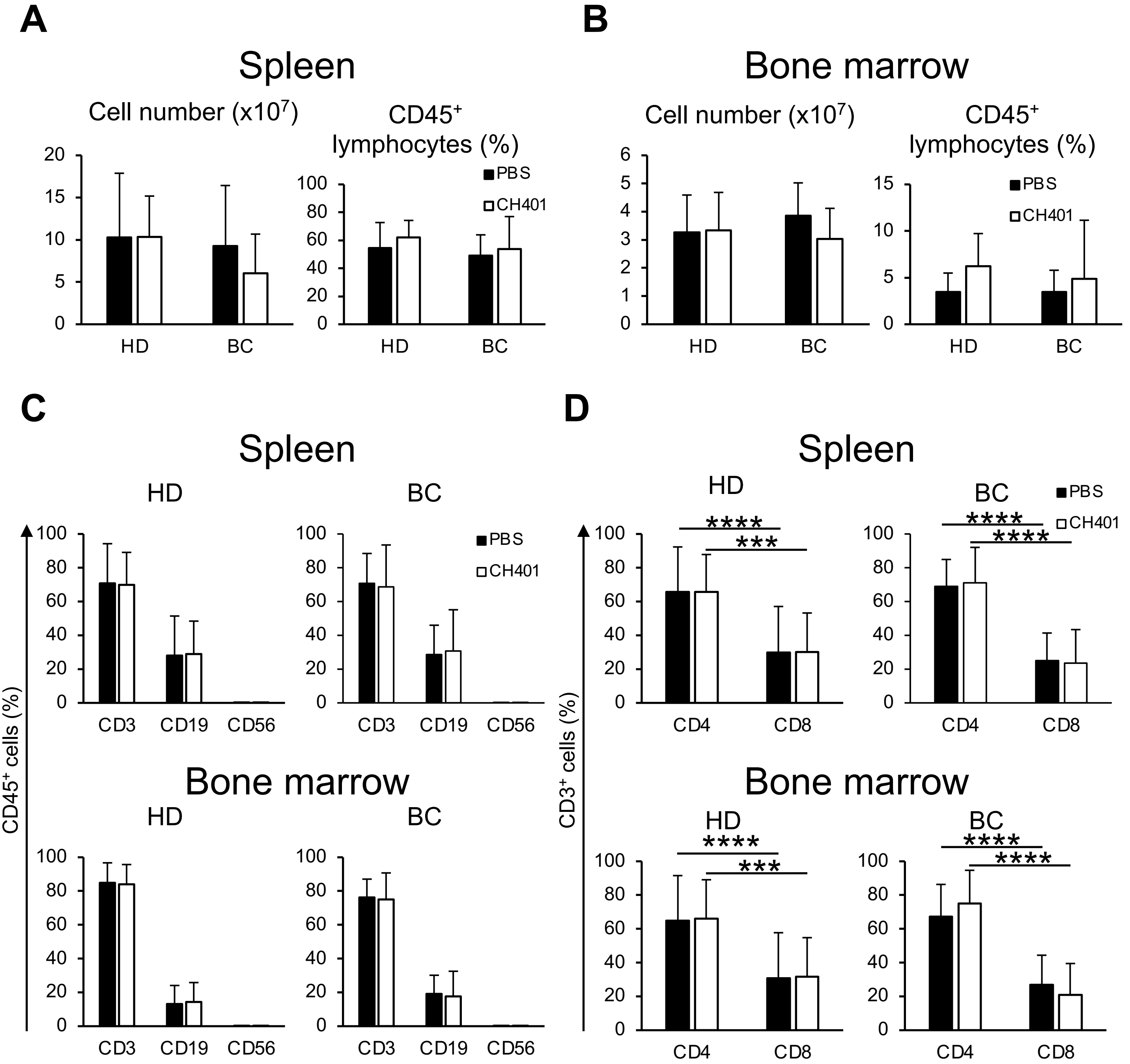

Figure S3 Proportions of the lymphocyte subsets localized in the spleens and the bone marrows transplanted with HD (PBS; n = 22, CH401MAP; n = 17) or BC (PBS; n = 12, CH401MAP; n = 14) PBMCs. PBS groups and CH401MAP groups are shown as black (PBS) or white (CH401MAP) bars. **(A-B)** Cell numbers and percentages of $CD45^+$ lymphocytes in the spleen (A) and the bone marrow (B). *p = 0.05. **(C)** Percentages of each lymphocyte subset ($CD3^+$ T cell, $CD19^+$ B cell, and $CD56^+$ NK cell) in the spleen and the bone marrow. **(D)** Percentages of $CD4^+$ and $CD8^+$ T cells in the spleen and the bone marrow. ****p = 0.0001.

Figure S4

A

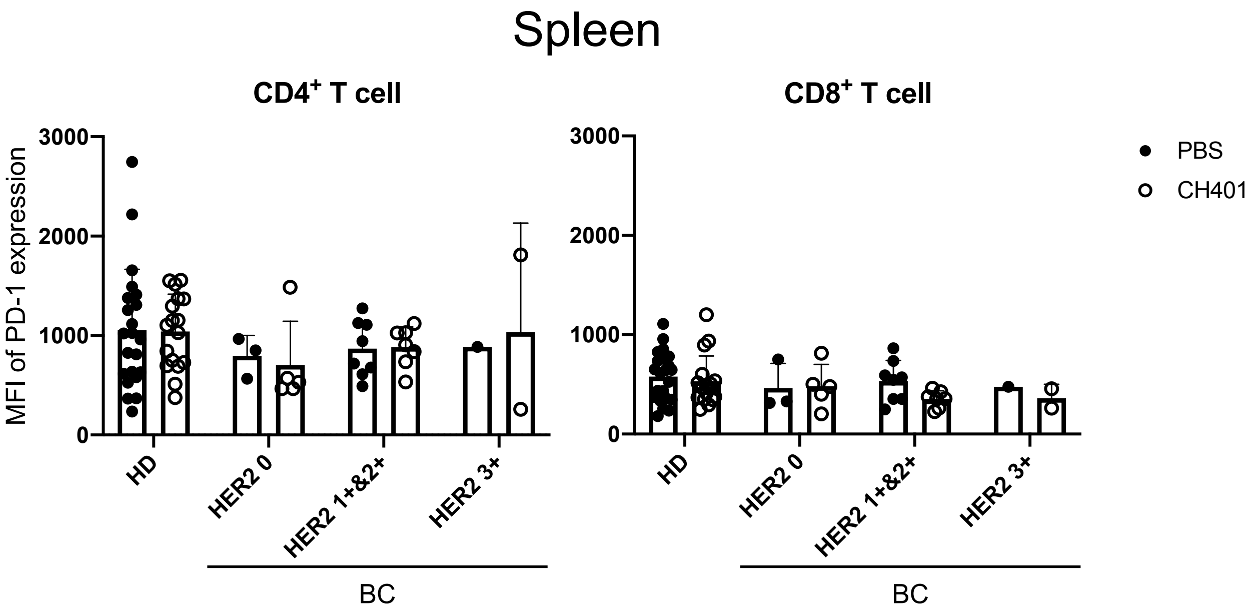

Spleen
CD4+ T cell
CD8+ T cell
MFI of PD-1 expression
3000
2000
1000
0
HD
HER2 0
HER2 1+&2+
HER2 3+
BC
PBS
CH401

B

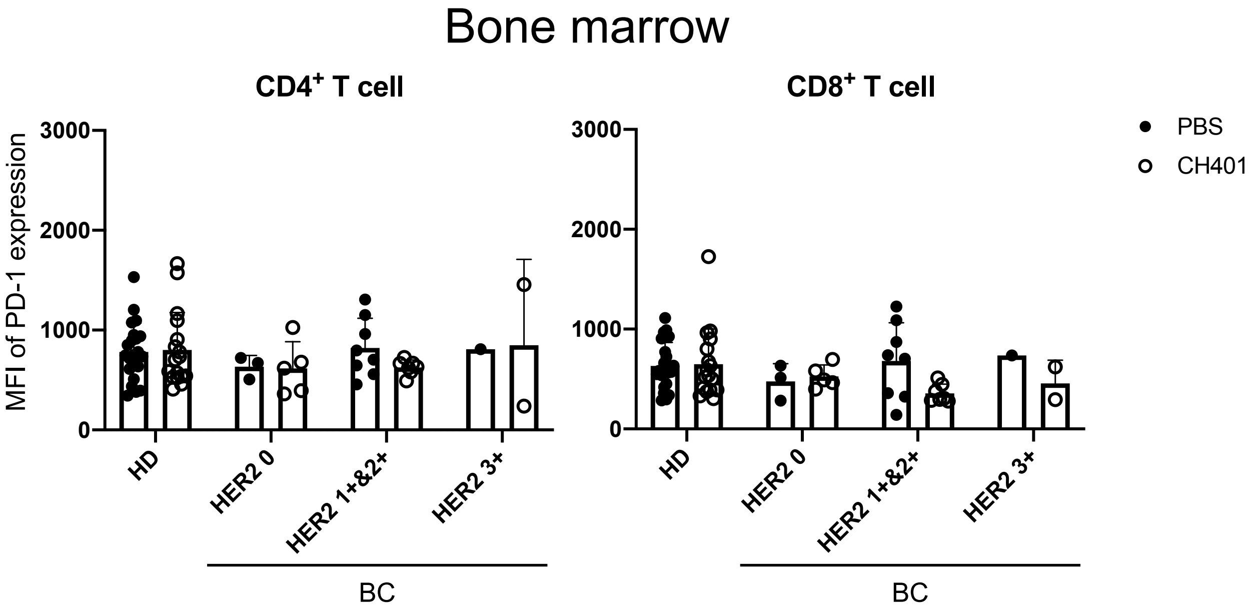

Bone marrow
CD4+ T cell
CD8+ T cell
MFI of PD-1 expression
3000
2000
1000
0
HD
HER2 0
HER2 1+&2+
HER2 3+
BC
PBS
CH401

C

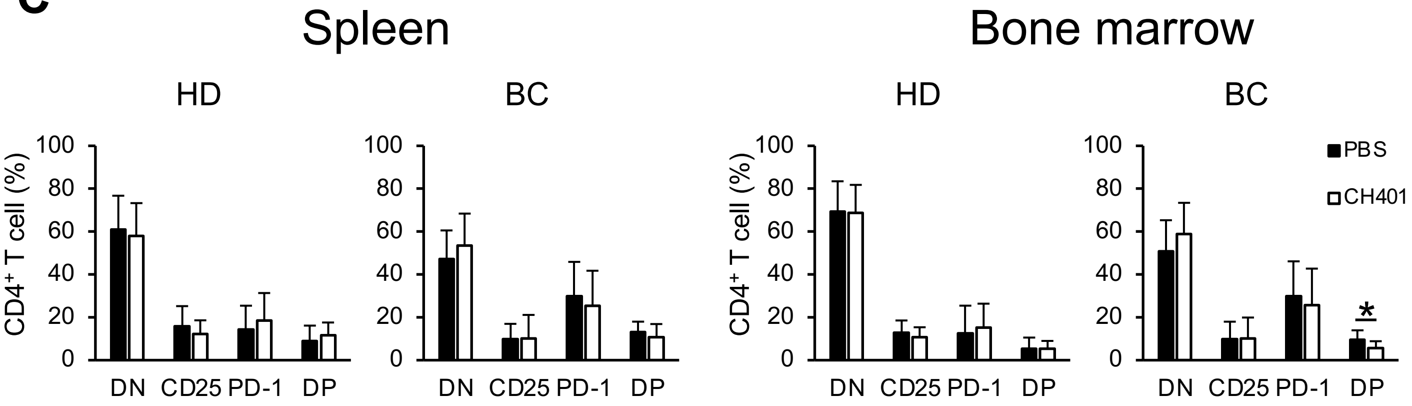

D

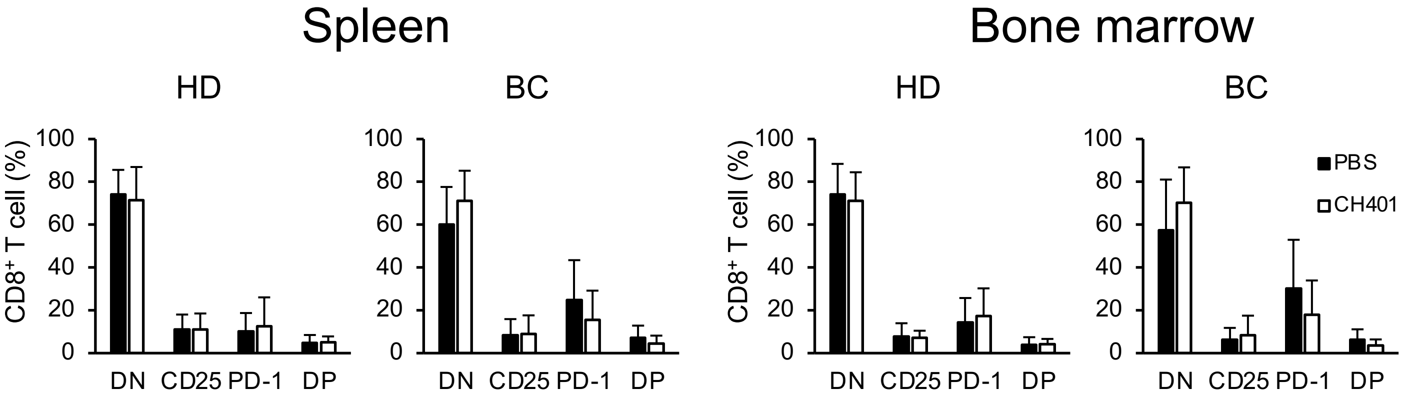

Figure S4 Effects of CH401MAP administration on the activation/exhaustion of engrafted human T cells. The expression of CD25 and PD-1 on human T cells was compared between the PBS- and CH401MAP-administrated spleen and bone marrow cells of HD-M (PBS; n = 22, CH401MAP; n = 17) or BC-M (PBS, HER2 0; n = 3, HER2 1+&2+; n = 8, HER2 3+; n = 1, CH401MAP, HER2 0; n = 5, HER2 1+&2+; n = 7, HER2 3+; n = 2). **(A-B)** MFI of PD-1 expression on $CD4^+$ T cells or $CD8^+$ T cells in the spleen (A) and the bone marrow (B) of hu-PBL hIL-4 NOG mice. **(C-D)** CD25 and PD-1 expression on $CD4^+$ T cells (C) and $CD8^+$ T cells (D) localized in the spleen and the bone marrow of HD-M or BC-M. Double-negative DN; $CD25^-PD\text{-}1^-$, CD25; $CD25^+PD\text{-}1^-$, PD-1; $CD25^-PD\text{-}1^+$, Double-positive DP; $CD25^+PD\text{-}1^+$. PBS and CH401MAP administration shown as black (PBS) or white (CH401MAP) bars. *p = 0.05. BC, breast cancer; HER2, Human epidermal growth factor receptor 2; HD, healthy donor; PBMCs, peripheral blood mononuclear cells; MFI, mean fluorescence intensity.

Figure S5

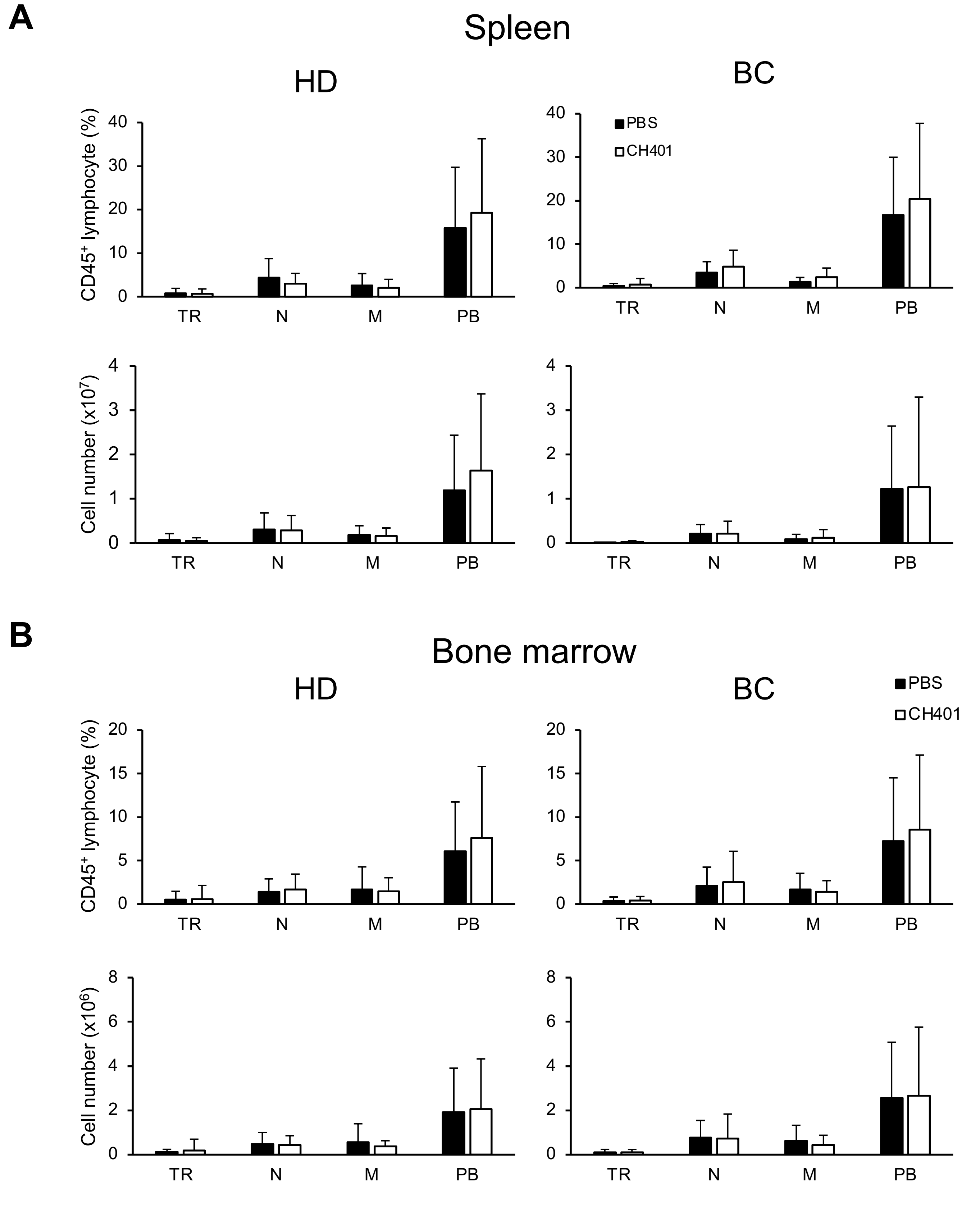

Figure S5 Frequency and cell number of B cell subsets. TR; Transitional, N; Naïve, M; Memory, PB; Plasmablast. The proportions in the spleens **(A)** and the bone marrows **(B)** transplanted with HD (PBS; *n* = 26, CH401MAP; *n* = 18) or BC (PBS; *n* = 13, CH401MAP; *n* = 19) PBMCs with mean ± SD of frequencies (upper) and cell numbers (lower) are shown.

Figure S6

A

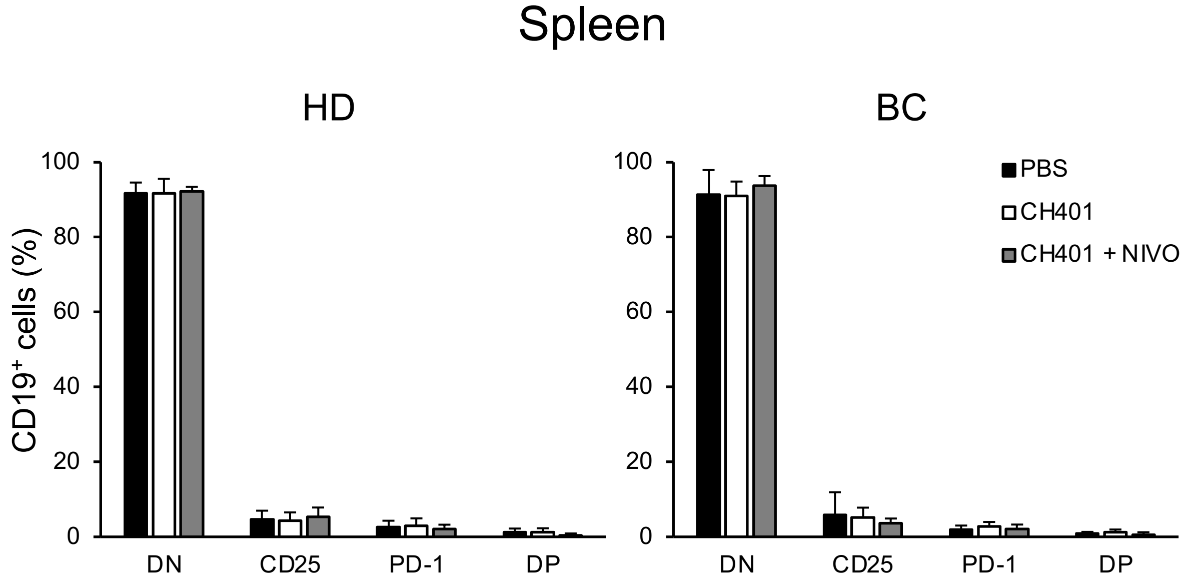

B

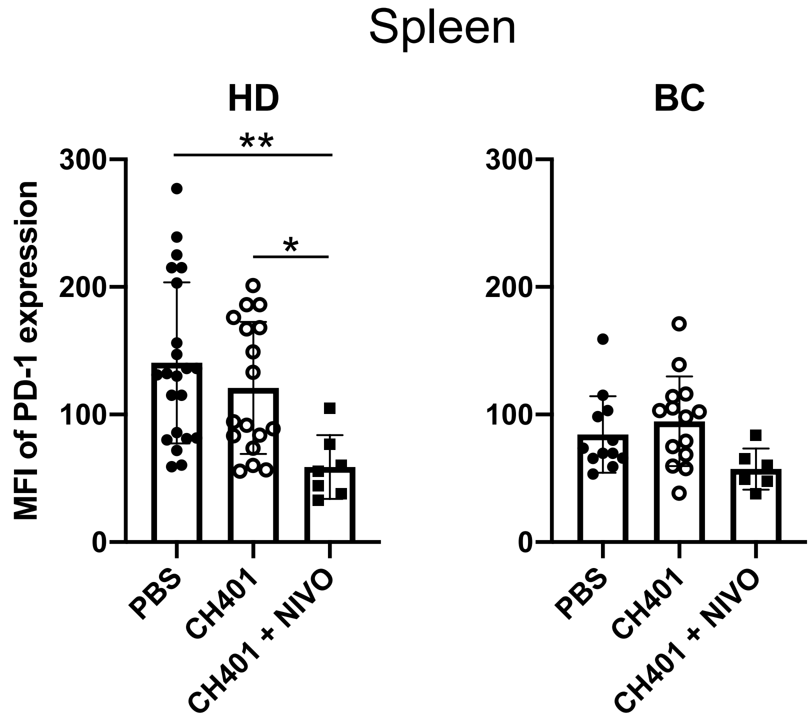

Figure S6 Expression of CD25 and PD-1 molecules localized in the spleens and the bone marrows transplanted with HD (PBS; *n* = 22, CH401MAP; *n* = 17, CH401MAP + NIVO; *n* = 7) or BC (PBS; *n* = 12, CH401MAP; *n* = 14, CH401MAP + NIVO; *n* = 6) PBMCs. **(A)** CD25 and PD-1 expressions in $CD19^+$ B cells localized in the spleens and the bone marrows of HD-M (left) or BC-M (right). Each cell subset, DN; $CD25^-PD\text{-}1^-$, CD25; $CD25^+PD\text{-}1^-$, PD-1; $CD25^-PD\text{-}1^+$, DP; $CD25^+PD\text{-}1^+$, are shown. **(B)** MFI of PD-1 expression on $CD19^+$ B cells in the spleens and the bone marrows. NIVO, Nivolumab.

Figure S7

A

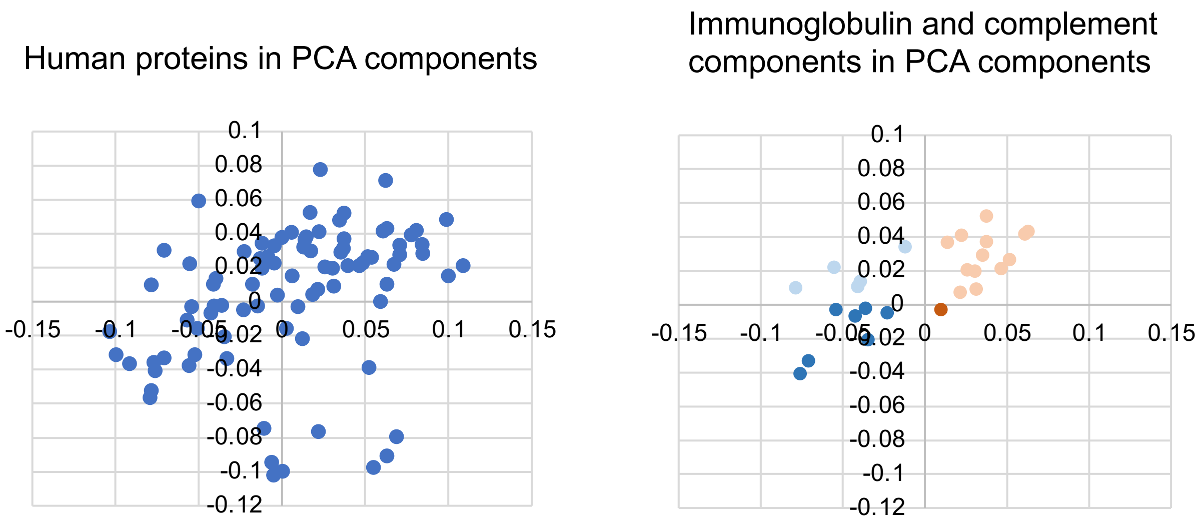

Human proteins in PCA components
Immunoglobulin and complement components in PCA components

B

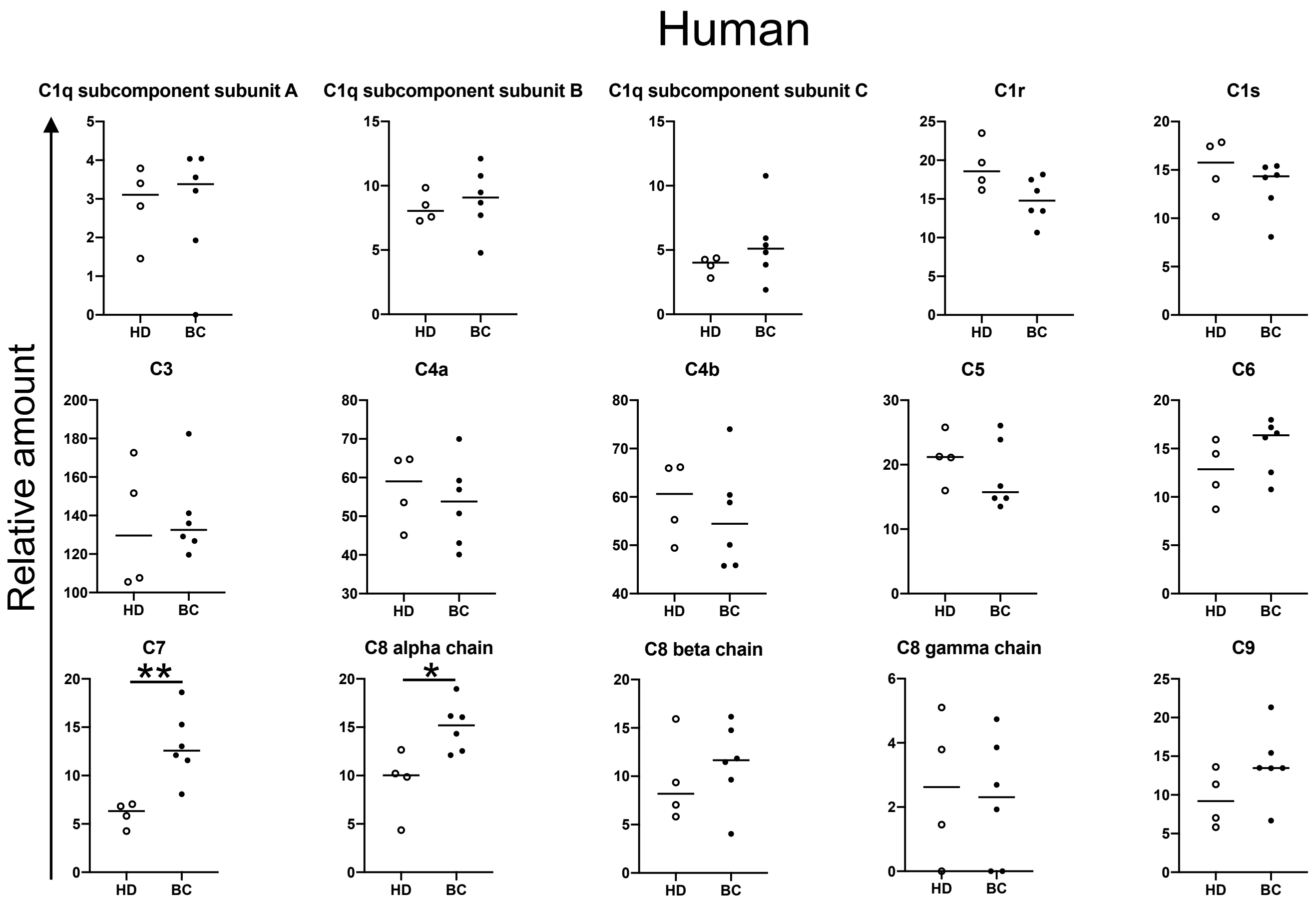

Human
Relative amount
C1q subcomponent subunit A
C1q subcomponent subunit B
C1q subcomponent subunit C
C1r
C1s
C3
C4a
C4b
C5
C6
C7
C8 alpha chain
C8 beta chain
C8 gamma chain
C9
HD
BC
**
*

C

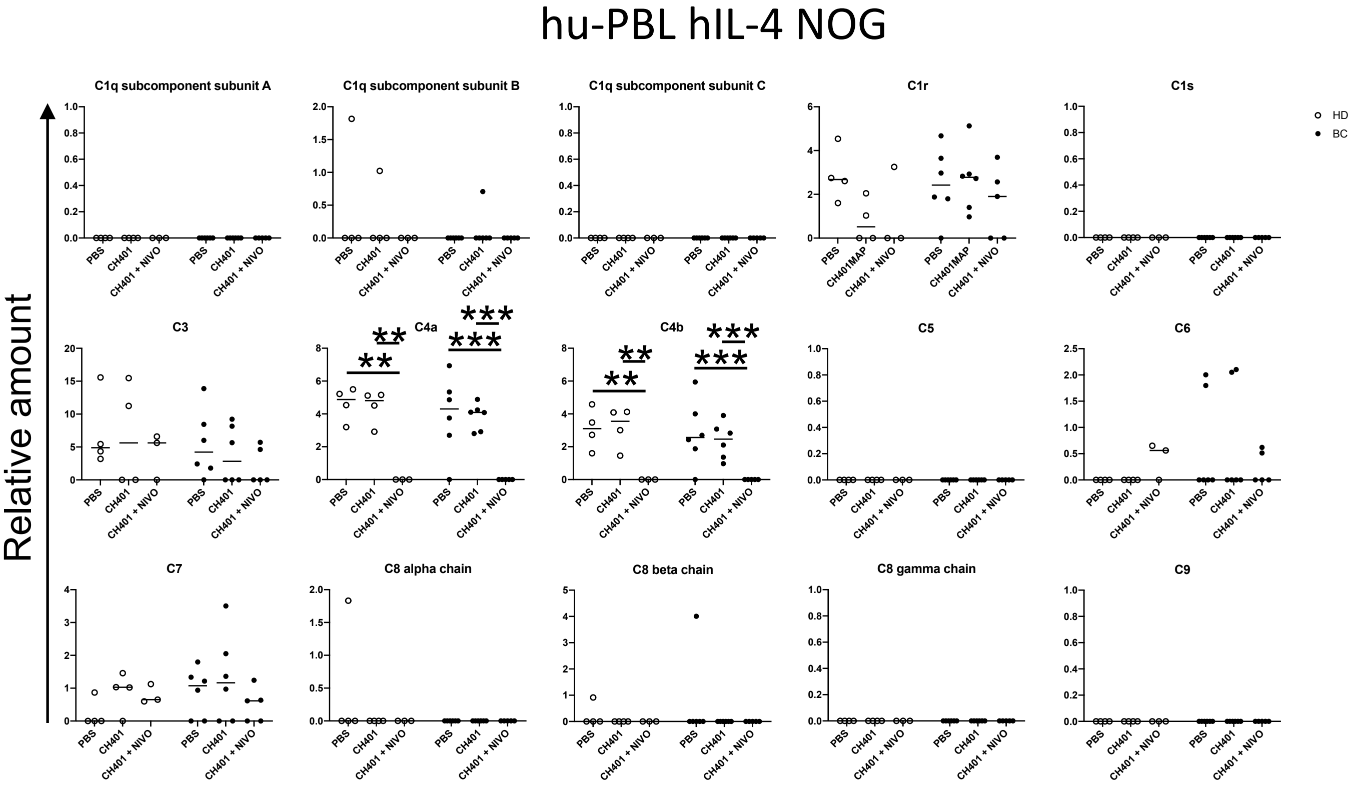

Figure S7 Comparison of proteins in the mouse plasma. **(A)** All the human proteins detected in the HD-M and BC-M were extracted from the results of the principal component analysis shown in Table S2 and shown in the left panel. Human immunoglobulin and complement proteins are extracted and shown in the right panel. The specific contents are shown in Table S2. Blue dots are sited in the left, and orange dots are in the right. **(B-C)** Relative amounts of the human complements in HD (open circle; *n* = 4) or BC (filled circle; *n* = 6) plasma (B) and HD-M (open circle; PBS; *n* = 4, CH401MAP; *n* = 4, CH401MAP + NIVO; *n* = 3) or BC-M (filled circle; PBS; *n* = 6, CH401MAP; *n* = 6, CH401MAP + NIVO; *n* = 5) (C). NIVO, Nivolumab.

Figure S8

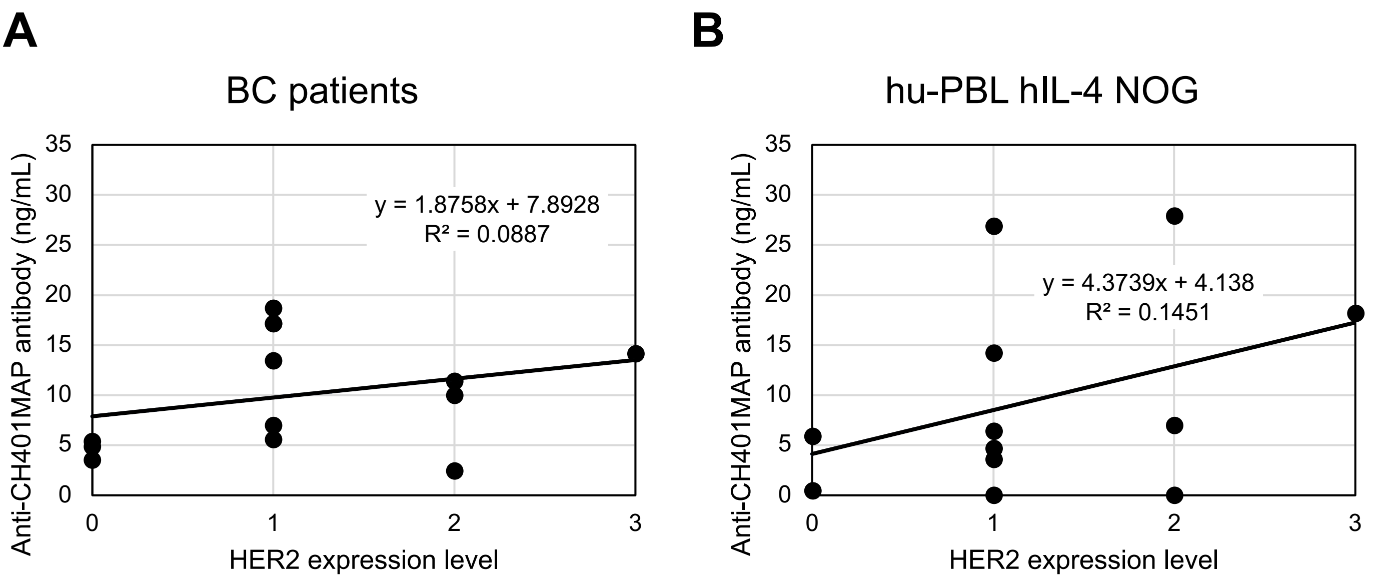

Figure S8 Correlation of donor HER2 expression level and concentration of anti-CH401MAP antibody in breast cancer patients (**A**; *n* = 13) and BC-M (**B**; *n* = 12) plasma. HER2, Human epidermal growth factor receptor 2.

Figure S9

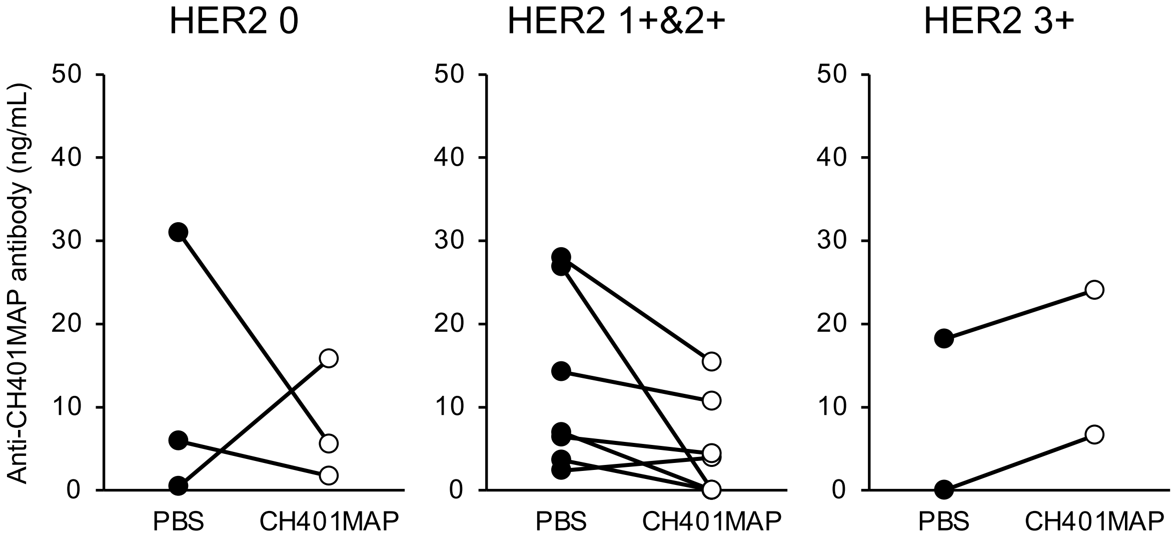

Figure S9 Concentrations of anti-CH401MAP antibody in plasma of BC-M.
Anti-CH401MAP antibody level was compared among subgroups (HER2 0, HER2 1+2+, HER2 3+) of the BC-M PBMCs (HER2 0; *n* = 3, HER2 1+&2+; *n* = 7, HER2 3+; *n* = 2). The concentration was compared between PBS (filled circle) or CH401MAP (open circle) groups, and the lines represent the mice transplanted with the same donor PBMC. HER2, Human epidermal growth factor receptor 2.

Figure S10

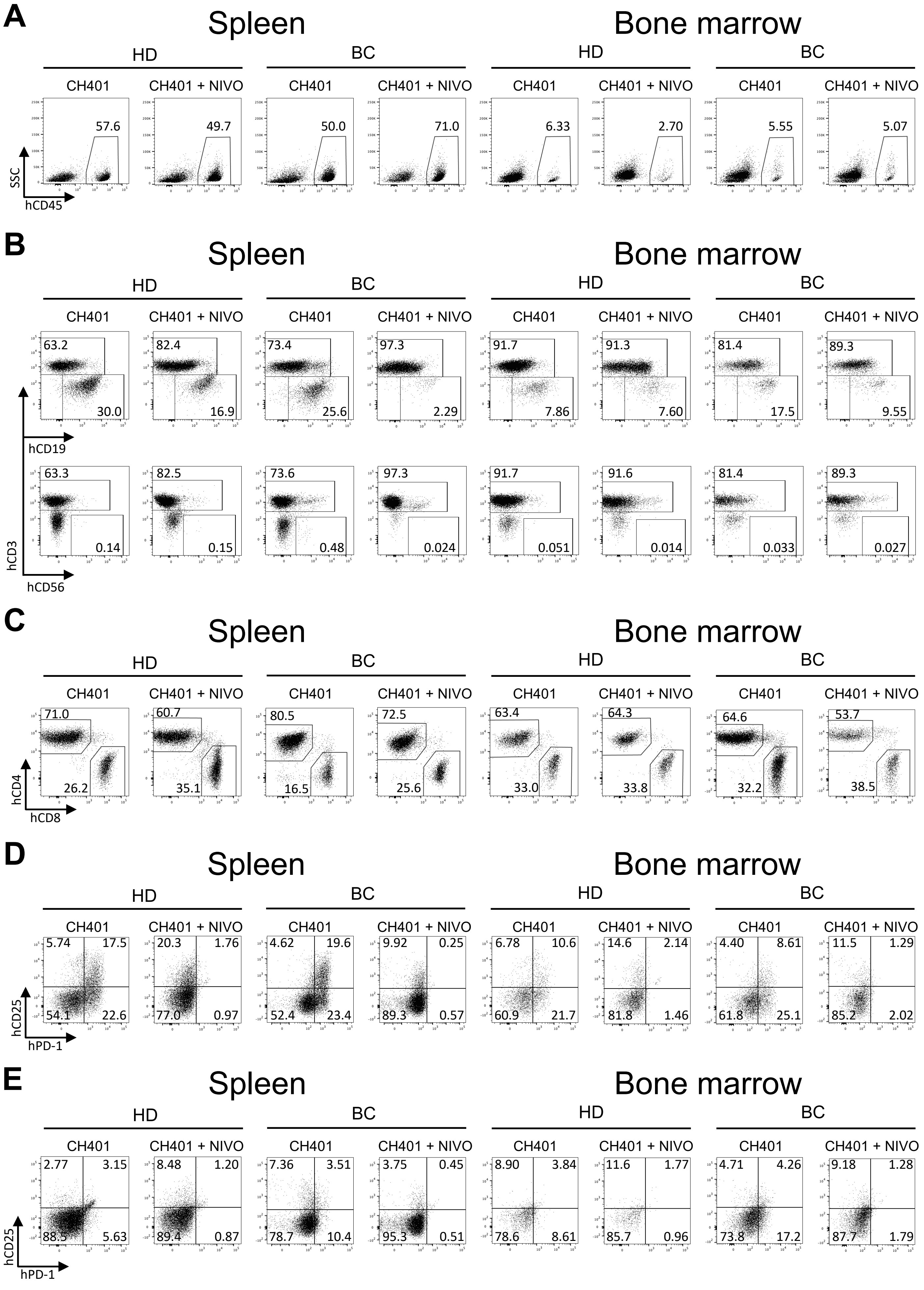
A
Spleen
Bone marrow
HD
BC
HD
BC
CH401
CH401 + NIVO
CH401
CH401 + NIVO
CH401
CH401 + NIVO
CH401
CH401 + NIVO
57.6
49.7
50.0
71.0
6.33
2.70
5.55
5.07
SSC
hCD45
B
Spleen
Bone marrow
HD
BC
HD
BC
CH401
CH401 + NIVO
CH401
CH401 + NIVO
CH401
CH401 + NIVO
CH401
CH401 + NIVO
63.2
30.0
82.4
16.9
73.4
25.6
97.3
2.29
91.7
7.86
91.3
7.60
81.4
17.5
89.3
9.55
hCD19
63.3
0.14
82.5
0.15
73.6
0.48
97.3
0.024
91.7
0.051
91.6
0.014
81.4
0.033
89.3
0.027
hCD3
hCD56
C
Spleen
Bone marrow
HD
BC
HD
BC
CH401
CH401 + NIVO
CH401
CH401 + NIVO
CH401
CH401 + NIVO
CH401
CH401 + NIVO
71.0
26.2
60.7
35.1
80.5
16.5
72.5
25.6
63.4
33.0
64.3
33.8
64.6
32.2
53.7
38.5
hCD4
hCD8
D
Spleen
Bone marrow
HD
BC
HD
BC
CH401
CH401 + NIVO
CH401
CH401 + NIVO
CH401
CH401 + NIVO
CH401
CH401 + NIVO
5.74
17.5
54.1
22.6
20.3
1.76
77.0
0.97
4.62
19.6
52.4
23.4
9.92
0.25
89.3
0.57
6.78
10.6
60.9
21.7
14.6
2.14
81.8
1.46
4.40
8.61
61.8
25.1
11.5
1.29
85.2
2.02
hCD25
hPD-1
E
Spleen
Bone marrow
HD
BC
HD
BC
CH401
CH401 + NIVO
CH401
CH401 + NIVO
CH401
CH401 + NIVO
CH401
CH401 + NIVO
2.77
3.15
88.5
5.63
8.48
1.20
89.4
0.87
7.36
3.51
78.7
10.4
3.75
0.45
95.3
0.51
8.90
3.84
78.6
8.61
11.6
1.77
85.7
0.96
4.71
4.26
73.8
17.2
9.18
1.28
87.7
1.79
hCD25
hPD-1

Figure S10 Typical patterns and gating of human lymphocytes in FCM used in Fig. 5 and Fig. 6. HD-M and BC-M spleens and bone marrows administrated with CH401MAP or CH401MAP + NIVO were analyzed. The gating strategies were shown in Fig. S1A. Percentages of the gated cells were shown in the panels. **(A)** Typical patterns of human $CD45^+$ cell engraftment. **(B)** Typical patterns of each human lymphocyte subset ($CD3^+$ T cells, $CD19^+$ B cells and $CD56^+$ NK cells.). **(C)** Typical patterns of $CD4^+$ and $CD8^+$ T cells. **(D)** Typical expression patterns of CD25 and PD-1 in $CD4^+$ T cells. **(E)** Typical expression patterns of CD25 and PD-1 in $CD8^+$ T cells. BC, breast cancer; HD, healthy donor; NIVO, nivolumab.

Figure S11

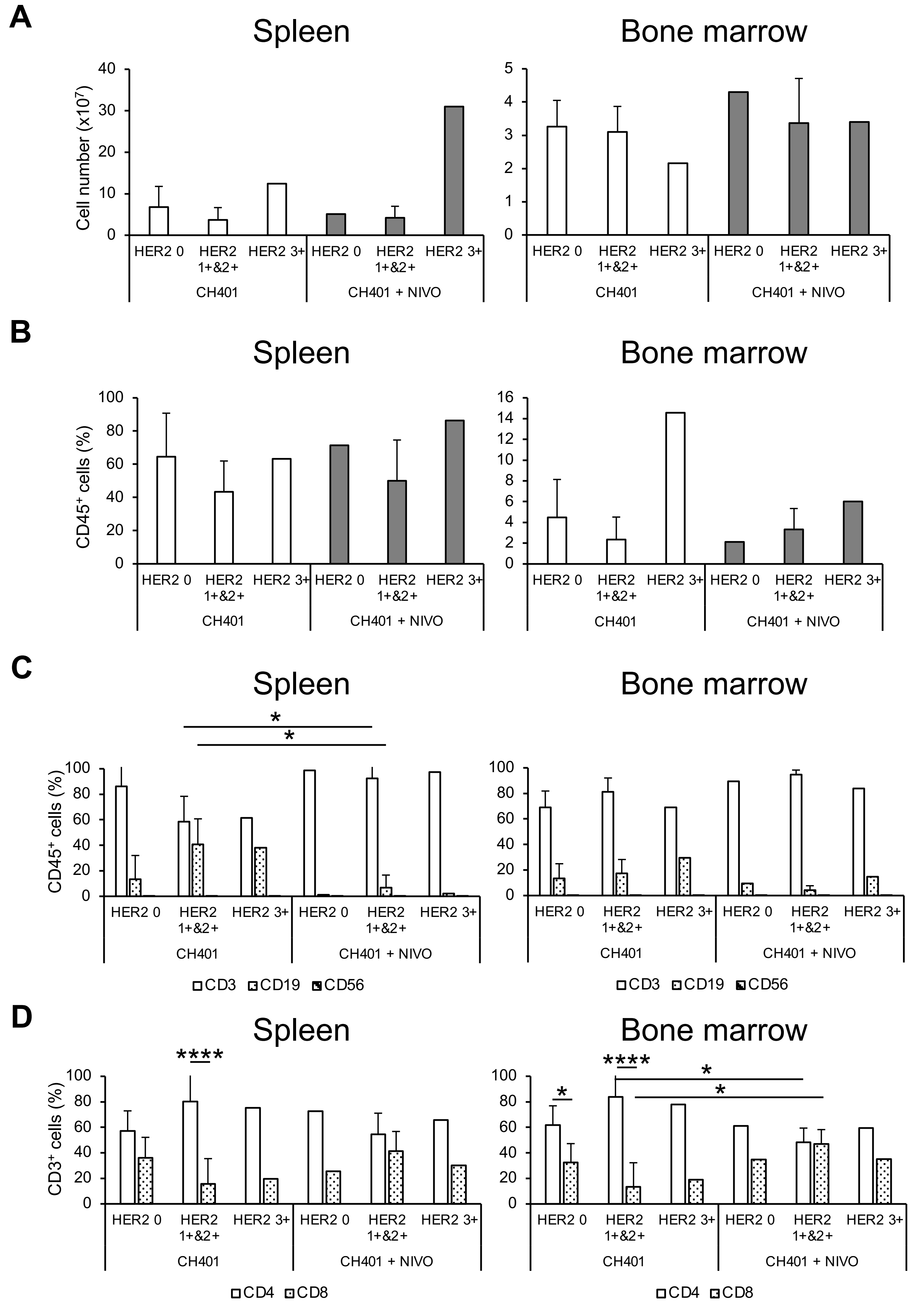
A
Spleen
Cell number (x10^7)
40
30
20
10
0
HER2 0
HER2 1+&2+
HER2 3+
CH401
CH401 + NIVO
Bone marrow
5
4
3
2
1
0
B
Spleen
CD45+ cells (%)
100
80
60
40
20
0
Bone marrow
16
14
12
10
8
6
4
2
0
C
Spleen
CD45+ cells (%)
Bone marrow
CD3
CD19
CD56
D
Spleen
CD3+ cells (%)
Bone marrow
CD4
CD8

Figure S11 Effects of nivolumab on the cellularity of BC-M subgroups (HER2 0, HER2 1+2+, HER2 3+). Cellularity of the lymphocytes of BC-M subgroups (HER2 0, HER2 1+2+, HER2 3+) with and without NIVO administration were examined for spleen and bone marrow cells. CH401MAP (HER2 0; $n = 5$, HER2 1+&2+; $n = 7$, HER2 3+; $n = 2$) and CH401MAP + NIVO (HER2 0; $n = 1$, HER2 1+&2+; $n = 3$, HER2 3+; $n = 2$). **(A)** Cell number of the spleen and the bone marrow. CH401MAP and CH401MAP + NIVO administrated mice were shown as white (CH401MAP) or gray bars (CH401MAP + NIVO). **(B)** Proportion of human $CD45^+$ cells. CH401MAP and CH401MAP + NIVO administrated mice were shown as white (CH401MAP) or gray bars (CH401MAP + NIVO). **(C)** Proportion of lymphocyte subsets ($CD3^+$ T cells, $CD19^+$ B cells and $CD56^+$ NK cells) were shown as each dot and bar. *$p = 0.05$, **$p = 0.01$. **(D)** Percentage of $CD4^+$ and $CD8^+$ T cells were shown as each dot bar. **$p = 0.01$. BC, breast cancer; HER2, Human epidermal growth factor receptor 2; HD, healthy donor; NIVO, Nivolumab.

Figure S12

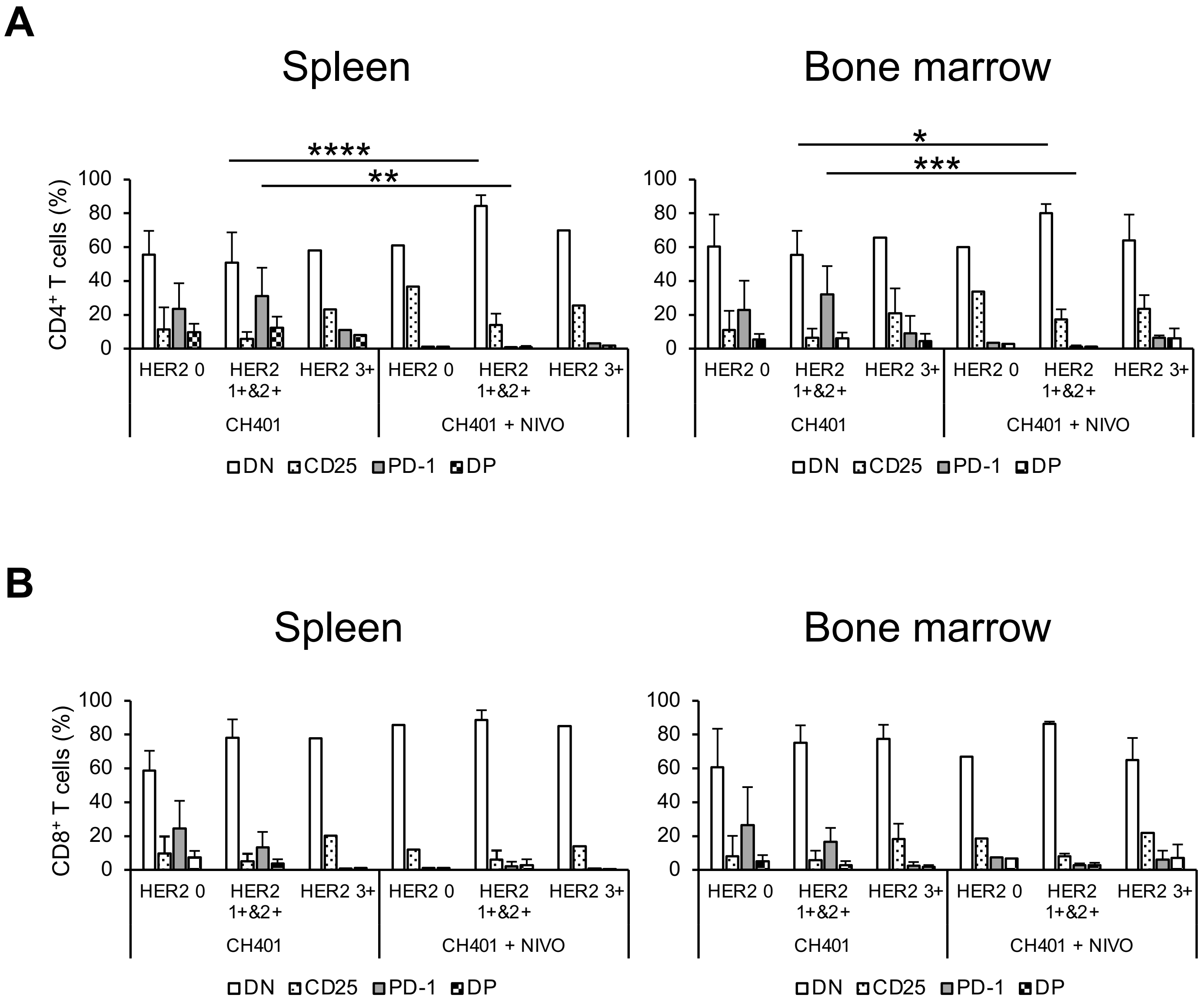
A
Spleen
Bone marrow
CD4⁺ T cells (%)
100
80
60
40
20
0
****
**
*
***
HER2 0
HER2 1+&2+
HER2 3+
CH401
CH401 + NIVO
DN
CD25
PD-1
DP
B
Spleen
Bone marrow
CD8⁺ T cells (%)
HER2 0
HER2 1+&2+
HER2 3+
CH401
CH401 + NIVO
DN
CD25
PD-1
DP

C

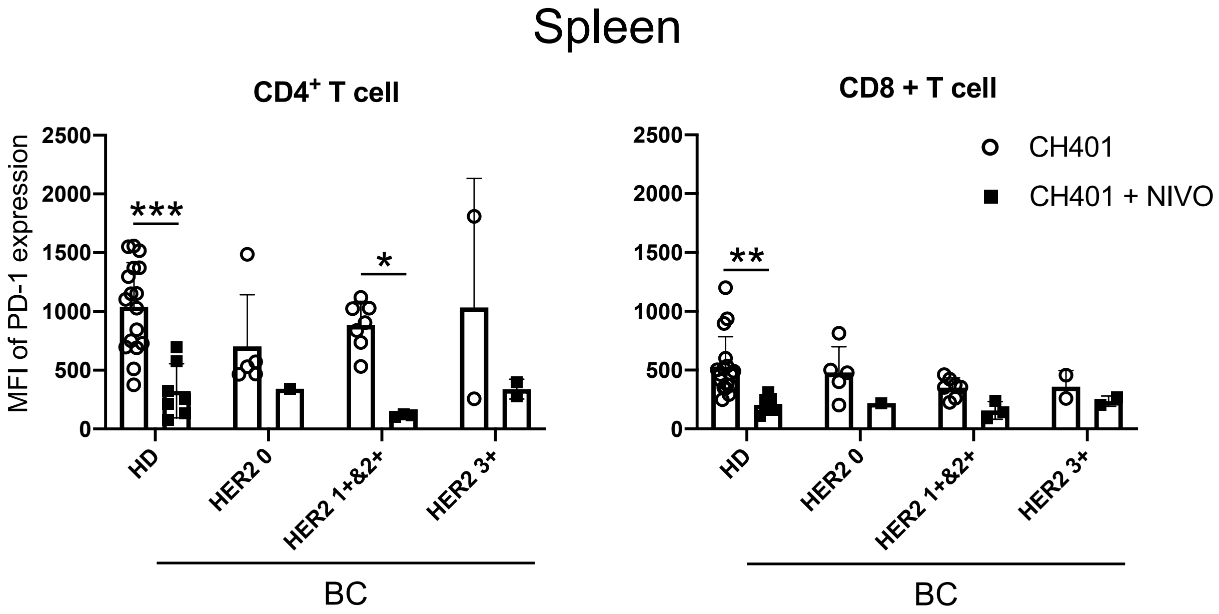

D

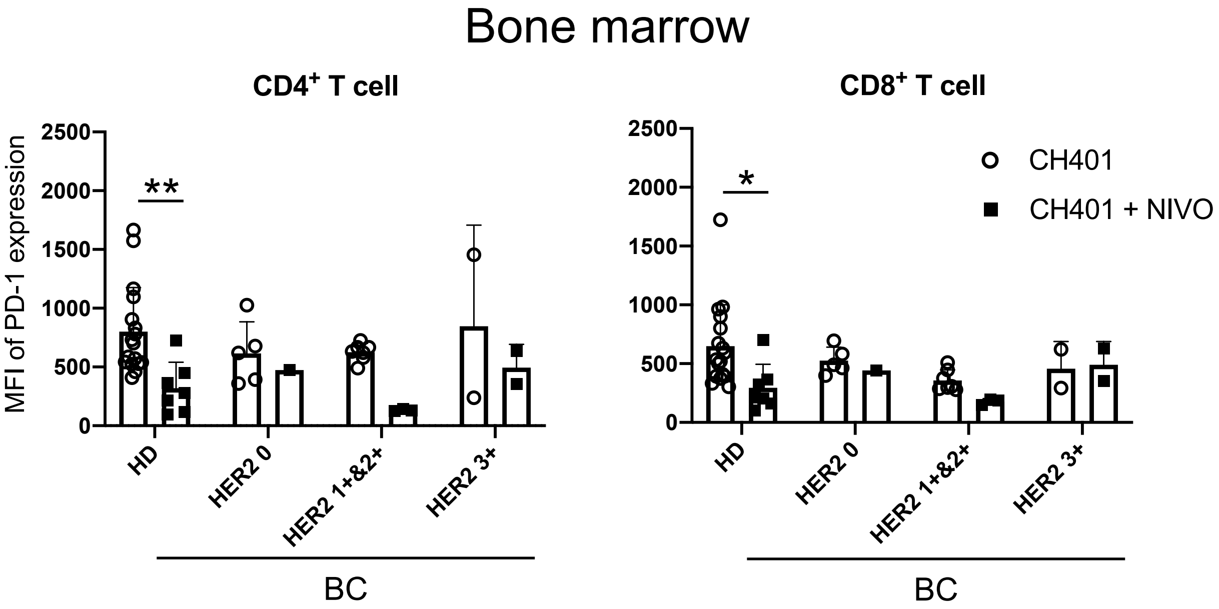

Figure S12 Nivolumab (NIVO) effects on the activation/exhaustion of engrafted human T cells of BC-M subgroups (HER2 0, HER2 1+2+, HER2 3+).

Proportions of the T cell subsets in BC-M subgroups (HER2 0, HER2 1+2+, HER2 3+) with and without NIVO administration were examined for spleen and bone marrow cells. CH401MAP (HER2 0; *n* = 5, HER2 1+&2+; *n* = 7, HER2 3+; *n* = 2) and CH401MAP + NIVO (HER2 0; *n* = 1, HER2 1+&2+; *n* = 3, HER2 3+; *n* = 2). **(A)** CD4$^+$ T cells and **(B)** CD8$^+$ T cells localized in the spleen and the bone marrow of BC-M. Double-negative DN; CD25$^-$PD-1$^-$, CD25; CD25$^+$PD-1$^-$, PD-1; CD25$^-$PD-1$^+$, Double-positive DP; CD25$^+$PD-1$^+$, were shown as each dot bar. *p = 0.05, **p = 0.01. **(C-D)** MFI of PD-1 expression on CD4$^+$ T cells or CD8$^+$ T cells in the spleen (C) and the bone marrow (D) of hu-PBL hIL-4 NOG mice. *p = 0.05, **p = 0.01, ****p = 0.0001. BC, breast cancer; HER2, Human epidermal growth factor receptor 2; HD, healthy donor; PBMCs, peripheral blood mononuclear cells; MFI, mean fluorescence intensity. NIVO, Nivolumab.

Table S1 List of fluorochrome-labeled antibodies

(A) Information of fluorochrome-labeled antibodies

Antibody	Clone	Dilution	Company
FITC anti-human CD3	UCHT1	1:1	BioLegend
APC anti-human CD4	RPA-T4	1:9	BioLegend
BrilliantViolet510™ anti-human CD4	OKT4	No	BioLegend
PE/Cy7 anti-human CD5	UCHT2	1:3	BioLegend
Alexa Fluor®700 anti-human CD8	HIT8a	No	BioLegend
Pacific Blue™ anti-human CD8a	RPA-T8	No	BioLegend
APC/Cy7 anti-human CD19	HIB19	1:1	BioLegend
PE anti-human CD25	BC96	1:1	BioLegend
PE/Cy7 anti-human CD25	BC96	1:1	BioLegend
PE anti-human CD27	M-T271	1:3	BD Bioscience
Alexa Fluor®700 anti-human CD38	HIT2	1:1	BioLegend
Pacific Blue™ anti-human CD45	HI30	1:9	BioLegend
BrilliantViolet650™ anti-human CD45RA	HI100	1:3	BioLegend
APC anti-human CD45RA	HI100	1:3	BioLegend
APC/Cy7 anti-human CD45RO	UCHL1	1:9	BioLegend
PE/Cy7 anti-human CD56	NCAM16.2	1:99	BD Bioscience
PE anti-human CD62L	DREG-56	1:9	BioLegend
BUV395 anti-human CD274 (PD-L1)	MIH1	1:3	BD Bioscience
PerCP/Cy5.5 anti-human CD279 (PD-1)	EH12.2H7	No	BioLegend
FITC anti-human IgD	IA6-2	1:1	BD Bioscience

(B) Address of the companies

Company	Address
BD Bioscience	1 Becton Drive, Franklin Lakes, NJ
BioLegend	9727 Pacific Heights Blvd, San Diego, CA
eBioscience	10255 Science Center Drive San Diego, CA

Table S2 PCA components involving Igs and complements

Var ID (Primary)	M1.p[4]	M1.p[2]
IGG1_HUMAN	0.037362	0.037317
IGHG2_HUMAN	0.035256	0.029402
IGHG3_HUMAN	0.02234	0.04121
IGHG4_HUMAN	0.025845	0.020523
HV108_HUMAN	0.051547	0.026522
HV315_HUMAN	0.021524	0.007315
HV323_HUMAN	0.030483	0.019763
KV320_HUMAN	0.03132	0.009194
IGLC2_HUMAN	0.013835	0.036759
C1QB_HUMAN	0.046394	0.021494
CO3_HUMAN	0.060945	0.041602
CO4A_HUMAN	0.063008	0.043199
CO4B_HUMAN	0.037345	0.052229
VINC_HUMAN (+3)	0.108976	0.021438
LDHA_HUMAN (+2)	0.100106	0.015284
FIBA_HUMAN	0.098853	0.048293
ALS_HUMAN	0.084489	0.028636
PSB1_HUMAN	0.084395	0.033704
MOES_HUMAN (+2)	0.080748	0.041933
PEDF_HUMAN	0.077671	0.03925
HGFA_HUMAN	0.070834	0.033347
APOE_HUMAN	0.070723	0.027693
HEP2_HUMAN	0.067322	0.022247
K1C9_HUMAN	0.063074	0.010539
ITIH2_HUMAN	0.062503	0.071281
PPAC_HUMAN (+5)	0.059179	0.000219
K2C1_HUMAN	0.053858	0.026402
KNG1_HUMAN	0.048657	0.022774
K1C10_HUMAN	0.039781	0.021344
ITIH1_HUMAN	0.037129	0.031681
PON1_HUMAN	0.034772	0.048185
FIBB_HUMAN	0.022941	0.077919
A1AT_HUMAN	0.018622	0.004281
FETUA_HUMAN	0.017499	0.029903
CFAH_HUMAN	0.016905	0.052689
CERU_HUMAN	0.014543	0.038458
ALBU_HUMAN	0.013107	0.032333
HGFL_HUMAN	0.006212	0.015415
HPT_HUMAN	0.005959	0.041062
HV307_HUMAN	0.009683	-0.00291
COR1A_HUMAN	0.002858	-0.01561
HBB_HUMAN (+2)	0.068687	-0.07937
ALDOC_HUMAN (+2)	0.062972	-0.09067
HBA_HUMAN (+2)	0.054916	-0.09743
APOB_HUMAN	0.052422	-0.03843
RAGP1_HUMAN	0.022069	-0.0761
ITIH4_HUMAN	0.012139	-0.02167
TERA_HUMAN (+2)	0.000543	-0.09965

Var ID (Primary)	M1.p[4]	M1.p[2]
IGHM_HUMAN	-0.039519	0.0137459
IGKC_HUMAN	-0.041001	0.0105951
IGE_HUMAN (+1)	-0.055326	0.022283
C1R_HUMAN	-0.011989	0.0343653
CO6_HUMAN	-0.078608	0.0100375
IBP3_HUMAN	-0.002491	0.00410602
CFAB_HUMAN	-0.004539	0.0328342
HABP2_HUMAN	-0.004542	0.0227692
HEMO_HUMAN	-0.00844	0.0264991
A2MG_HUMAN	-0.012001	0.0199824
THRB_HUMAN	-0.012971	0.0254863
PRG4_HUMAN	-0.017688	0.0104115
PLMN_HUMAN	-0.022506	0.0296255
FIBG_HUMAN	-0.049931	0.059537
MGAL_HUMAN	-0.070862	0.0304332
PGRP2_HUMAN	-6.57E-05	0.0379723
IGJ_HUMAN	-0.034735	-0.0204294
IGHA1_HUMAN	-0.042578	-0.0065513
IGA2_HUMAN	-0.076159	-0.0406068
IGK_HUMAN	-0.022888	-0.0048357
KV311_HUMAN (+1)	-0.054138	-0.0028519
KV315_HUMAN	-0.070802	-0.0329065
CO7_HUMAN	-0.03619	-0.0020258
PSA1_HUMAN (+2)	-0.076922	-0.0356268
MYH9_HUMAN	-0.005066	-0.101863
6PGD_HUMAN (+1)	-0.006282	-0.0943581
NLRC4_HUMAN	-0.010666	-0.0744641
LYAM1_HUMAN (+2)	-0.014668	-0.0023355
G3P_HUMAN	-0.03315	-0.0333964
VTDB_HUMAN	-0.040919	-0.002274
KPYM_HUMAN	-0.050683	-0.0154031
TITIN_HUMAN	-0.052337	-0.0309784
APOC1_HUMAN	-0.055736	-0.0373865
PLSL_HUMAN	-0.056781	-0.0107038
MOES_HUMAN	-0.078489	-0.0521496
FLNA_HUMAN	-0.079066	-0.0560894
ALDOA_HUMAN	-0.091458	-0.0363648
PGAM1_HUMAN (+3)	-0.099717	-0.0310637
H2A1B_HUMAN (+29)	-0.103329	-0.0175759

*Colored data is shown as immunoglobulin or complement components in Fig S7A.

Table S3 Yield of hybridomas from HD-M/BC-M spleen cells.

Donor		Anti-CH401MAP antibody concentration of donor plasmas (ng/mL)	Administrated antigen	Mean number of colonies per well	Anti-CH401MAP antibody concentration in the mouse plasmas	% of positive well: human IgG antibody	% of positive well: Anti-CH401MAP antibody
HD	#1	12.00	PBS	0.7	6.61	49.4	3.6
			CH401MAP	2.2	14.50	96.9	22.5
	#2	8.87	PBS	4.6	3.44	100.0	30.2
			CH401MAP	8.8	0.48	100.0	80.2
	#3	12.37	PBS	4.3	5.51	100.0	26.0
			CH401MAP	8.3	11.74	100.0	79.2
	#4	4.97	PBS	8.3	12.89	100.0	1.0
			CH401MAP	10.7	0.47	100.0	2.1
			CH401MAP + NIVO	4.0	0.05	100.0	0.0
	#5	7.70	PBS	0.6	34.43	100.0	0.0
			CH401MAP	2.2	15.82	100.0	1.0
			CH401MAP + NIVO	3.3	0.36	71.9	0.0
BC	#1	3.55	PBS	8.3	27.93	100.0	60.4
			CH401MAP	19.6	15.43	100.0	100.0
	#2	11.38	PBS	5.4	30.96	57.8	85.9
			CH401MAP	20.8	3.35	100.0	100.0
	#3	2.47	PBS	8.6	0.00	67.7	100.0
			CH401MAP	4.6	0.00	36.5	94.8
			CH401MAP + NIVO	1.4	0.00	30.2	79.2
	#4	947.00	PBS	5.8	2.01	100.0	5.2
			CH401MAP	2.7	1.61	100.0	2.1
			CH401MAP + NIVO	4.1	1.09	5.2	0.0
	#5	20.20	PBS	0.7	0.29	1.0	0.0
			CH401MAP	1.4	2.82	100.0	0.0
			CH401MAP + NIVO	2.0	1.35	100.0	0.0
			CH401MAP + NIVO	1.6	4.73	67.7	0.0
